# A Computational Analysis of Abnormal Belief Updating Processes and Their Association With Psychotic Experiences and Childhood Trauma in a UK Birth Cohort

**DOI:** 10.1016/j.bpsc.2021.12.007

**Published:** 2022-07

**Authors:** Jazz Croft, Christoph Teufel, Jon Heron, Paul C. Fletcher, Anthony S. David, Glyn Lewis, Michael Moutoussis, Thomas H.B. FitzGerald, David E.J. Linden, Andrew Thompson, Peter B. Jones, Mary Cannon, Peter Holmans, Rick A. Adams, Stan Zammit

**Affiliations:** a Centre for Academic Mental Health, Population Health Sciences, Bristol Medical School, University of Bristol, Bristol, United Kingdom; b Cardiff University Brain Research Imaging Centre, School of Psychology, Cardiff University, Cardiff, United Kingdom; c Medical Research Council Centre for Neuropsychiatric Genetics and Genomics, Division of Psychological Medicine and Clinical Neurosciences, School of Medicine, Cardiff University, Cardiff, United Kingdom; d Cambridgeshire and Peterborough NHS Foundation Trust, Cambridge, United Kingdom; e Institute of Metabolic Science, University of Cambridge, Cambridge Biomedical Campus, Cambridge, United Kingdom; f Department of Psychiatry, University of Cambridge, Cambridge, United Kingdom; g University College London Institute of Mental Health, Division of Psychiatry, University College London, London, United Kingdom; h Wellcome Centre for Human Neuroimaging, Institute of Neurology, University College London, London, United Kingdom; i Centre for Medical Image Computing and AI, University College London, London, United Kingdom; j Max Planck-UCL Centre for Computational Psychiatry and Ageing Research, London, United Kingdom; k School of Psychology, University of East Anglia, Norwich, United Kingdom; l Warwick Medical School, University of Warwick, Warwick, United Kingdom; m Department of Psychiatry, Royal College of Surgeons in Ireland, Dublin, Ireland; n School for Mental Health and Neuroscience, Department of Psychiatry and Neuropsychology, Maastricht University, Maastricht, The Netherlands; o Orygen, The Centre for Youth Mental Health, University of Melbourne, Melbourne, Victoria, Australia

**Keywords:** ALSPAC, Belief updating, Childhood trauma, Cognition, Computational psychiatry, Psychosis

## Abstract

**Background:**

Psychotic experiences emerge from abnormalities in perception and belief formation and occur more commonly in those experiencing childhood trauma. However, which precise aspects of belief formation are atypical in psychosis is not well understood. We used a computational modeling approach to characterize belief updating in young adults in the general population, examine their relationship with psychotic outcomes and trauma, and determine the extent to which they mediate the trauma-psychosis relationship.

**Methods:**

We used data from 3360 individuals from the Avon Longitudinal Study of Parents and Children birth cohort who completed assessments for psychotic outcomes, depression, anxiety, and two belief updating tasks at age 24 and had data available on traumatic events assessed from birth to late adolescence. Unadjusted and adjusted regression and counterfactual mediation methods were used for the analyses.

**Results:**

Basic behavioral measures of belief updating (draws-to-decision and disconfirmatory updating) were not associated with psychotic experiences. However, computational modeling revealed an association between increased decision noise with both psychotic experiences and trauma exposure, although <3% of the trauma–psychotic experience association was mediated by decision noise. Belief updating measures were also associated with intelligence and sociodemographic characteristics, confounding most of the associations with psychotic experiences. There was little evidence that belief updating parameters were differentially associated with delusions compared with hallucinations or that they were differentially associated with psychotic outcomes compared with depression or anxiety.

**Conclusions:**

These findings challenge the hypothesis that atypical belief updating mechanisms (as indexed by the computational models and behavioral measures we used) underlie the development of psychotic phenomena.

SEE COMMENTARY ON PAGE 633

Psychotic experiences occur in about 5% to 10% of the population (1,2). They reflect a psychosis continuum that extends from mild and transient subclinical experiences to severe psychotic disorders such as schizophrenia (1,3). Childhood trauma is associated with an increased risk of psychotic outcomes (4, 5, 6, 7), but how trauma leads to psychosis is not well understood (6,8, 9, 10). Abnormalities in belief updating have been postulated as a mediating mechanism (11,12), but this hypothesis has not been empirically evaluated.

A classic hypothesis is that people with delusions reach decisions based on less information, referred to as the jumping to conclusions (JTC) bias (13, 14, 15). Delusion-prone individuals also tend to show an overreadiness to update beliefs in the face of disconfirmatory information, called the overadjustment bias (14,16,17), although in the context of certain social judgments, the opposite—a bias against disconfirmatory evidence—is seen (18,19). Numerous mechanisms can underlie each of these behavioral measures, and a greater understanding of the underlying processes might help reconcile apparent contradictions across tasks. For example, the JTC bias may be due to overweighting of evidence or a greater subjective cost of gathering more information, and an overadjustment bias could reflect greater updating to all evidence or just to unexpected evidence (20). Computational modeling methods are required to provide a detailed characterization of distinct mechanisms that underlie group differences in behavioral measures.

Computational models have been used to analyze belief-updating mechanisms in clinical or relatively small general population samples (20, 21, 22, 23, 24) but not in large population-based cohorts. Some, but not all (24,25), clinical studies that have adopted computational modeling methods have found overweighting of disconfirmatory evidence in people with psychosis (22), leading to belief instability (20) and greater inconsistency in decision making (23). While important, clinical case-control studies are particularly vulnerable to biases in estimating causal effects owing to reverse causation and confounding by factors such as IQ (26) or socioeconomic differences (21). Furthermore, it is unclear whether abnormal belief updating mechanisms are specific to psychosis, given that similar abnormalities have been reported in people with depression (20). Finally, atypical belief updating has been hypothesized as a mediating mechanism by which trauma exposure leads to psychotic symptoms, with trauma triggering the neurophysiological changes that underpin atypical cognitive processes (12,27,28). To date, however, no study has empirically examined this hypothesis.

Here, we used computational models to characterize distinct components of belief updating in adulthood and examined their association with trauma experienced from early childhood onward and psychotic experiences experienced in adulthood in a large population-based cohort. Our aims were to assess 1) whether specific components of abnormal belief updating are associated with trauma and psychotic experiences independently of cognitive, social, or genetic confounders; 2) whether associations are stronger for delusions than for hallucinations, as previously hypothesized (12,27), and whether they are specific to psychotic experiences rather than psychopathology more broadly; and 3) the extent to which abnormal belief updating might mediate the association between childhood trauma and psychotic experiences.

## Methods and Materials

### Sample

We used data from the Avon Longitudinal Study of Parents and Children (ALSPAC) (see http://www.bristol.ac.uk/alspac/researchers/our-data/ for a fully searchable data dictionary). Women residents in Avon, United Kingdom, with expected delivery dates between April 1, 1991, and December 31, 1992, were invited to take part in the study. Initially, 14,541 pregnancies were enrolled, resulting in 14,676 fetuses, 14,062 live births, and 13,988 alive children at 1 year of age (29, 30, 31). Ethical approval was obtained from the ALSPAC Ethics and Law Committee and the local research ethics committees. Our sample entailed 3360 participants with complete data on the draws-to-decision (DTD) task, mental health outcomes, and confounders, and 3369 participants for the probability estimation task (Figure S1).

### Measures

#### Psychotic Experiences

Psychotic experiences were assessed at approximately age 24 years using the Psychosis-like Symptoms Semi-structured Interview (2,32), which follows the definitions and rating rules of the Schedule for Clinical Assessment in Neuropsychiatry. Interviewers assessed the presence of 13 psychotic experiences (including hallucinations, delusions, and experiences of thought interference). Our primary outcome was the presence of suspected or definite psychotic experiences occurring at least monthly or reported as being distressing in the past year. As secondary outcomes we also examined 1) the subset meeting criteria for an at-risk mental state or psychotic disorder (2,32), 2) hallucinations and delusions separately, and 3) an ordinal measure of number of experiences as a measure of severity (0, 1, 2, 3+). See the Supplement for more details on this and other measures.

#### Anxiety and Depression

Anxiety (generalized anxiety disorder, panic disorder, social phobia, and specific phobias) and depression (moderate or severe depressive disorder) were assessed at age 24 years using the revised Clinical Interview Schedule, allowing derivation of ICD-10 diagnoses (33).

#### Childhood/Adolescent Trauma

A measure of exposure to trauma was derived from multiple assessments completed contemporaneously by the child or their parents from early childhood onward and one assessment at age 22 to supplement information on childhood sexual abuse. The number of types of trauma exposure (bullying; sexual, physical, or emotional abuse; emotional neglect) between ages 0 and 17 years (0, 1, 2, 3+) was used to index the dose of trauma exposure (5). For further details, see the Supplement.

#### Belief Updating Tasks

Participants completed two computerized tasks approximately at age 24, which were presented using custom-written MATLAB code (R2015a; The MathWorks, Inc.) with the Psychophysics Toolbox (34) and REDCap (35). To analyze performance in both tasks (DTD task and probability estimation task), we used basic behavioral measures and parameters derived from computational models.

In the literature, the DTD task is a standard means of assessing the JTC bias: participants request up to 10 beads, drawn with replacement, from one of two (hidden) jars, each with an 80:20 ratio of different colored beads, to decide from which of the two jars the beads were drawn. Participants completed five blocks of this task.

In line with previous literature, we derived two behavioral indices from participants’ performance: the average number of beads drawn before a decision was reached across the five blocks (labeled DTD) and the JTC bias (13,15), defined as an average DTD of two beads or fewer across the five blocks.

We also used a costed Bayesian model to derive two computational parameters underlying task performance (23,25): 1) cost of sampling, the subjective cost of requesting further information, with increasing cost leading to fewer draws, and 2) decision noise, the consistency of participants’ behavior given the sequences they saw and their cost of sampling. The role that these parameters play in the model is detailed in the Supplement. Briefly, the model assumes that subjects update their beliefs about the jars in a Bayes optimal fashion, which means that the point when they decide which jar is the source (DTD) is determined by their subjective cost of sampling more beads (itself defined relative to the subjective cost of being wrong) and inherent noisiness in their decision making.

For the probability estimation task, participants were presented with the same jars of beads as in the DTD task but were required to rate the probability, on a sliding scale, that each bead presented in a sequence of 30 beads was drawn from one jar or the other. Participants were informed that the jar from which the beads were drawn might change during the task. For all participants, this change happened after the 15th bead.

A basic behavioral measure of disconfirmatory or contrary updating (20,36) (overadjustment bias) was derived from the data, calculated as the size of belief updating after seeing a bead of a different color from the previous two or more identically colored beads (e.g., a blue bead after two or more red beads).

Moreover, the richer data generated by the probability estimation task permitted the use of a more complex model, the retrospective inference hidden Markov model (Figure S2) (37), to derive five parameters: 1) reversal probability, *r* (participants’ subjective probability that the jars switch on a given trial); 2) adjustment rate, *a* (the extent to which subjects’ estimates of jar probabilities are adjusted after drawing a bead, similar to a learning rate); 3) confidence in *r* (a Dirichlet parameter determining subjects’ willingness to update their initial estimate of reversal probability during the task); 4) window length, *L* (the number of previous trials used to re-estimate the reversal probability online); and 5) response noise, *1/ν* (the consistency of the subjects’ reported estimates with their models’ predictions). This treats the task as not just an inference problem (inferring which jar is most likely on each trial) but also a parameter learning problem (learning whether changes in jars were more or less likely than the subject originally anticipated).

For both tasks, parameters with non-normal distributions were transformed or collapsed into categorical measures when examining these as outcomes. Sensitivity analyses for collapsed variables were conducted using different cutoffs. See the Supplement for more details on tasks and parameters and Table S1 for correlations between them.

#### Confounders

Based on previous literature, we examined the following potential confounders: 1) cognitive functioning (IQ, working memory, and executive functioning at age 8 years), 2) socioeconomic status (maternal education, average household income, social class, crowding index) measured around birth, and 3) polygenic risk for schizophrenia (38) (see the Supplement for details).

### Statistical Analysis

Data analysis was completed in STATA version 15.2 (StataCorp LLC). We used logistic, ordinal, multinomial, and linear regression to estimate effect sizes (odds ratios [ORs] or beta coefficients), 95% CIs, and Wald test two-sided *p* values before and after adjusting for confounding. Nonlinear effects of exposures were examined by adding quadratic terms to the models. We used multivariate probit modeling to jointly model separate outcomes. Counterfactual mediation analysis was used to examine the extent to which belief updating (age 24) mediated the association between trauma (ages 0–17) and psychotic experiences (age 24), using the PARAMED command. While the cross-sectional mediator and outcome data makes it difficult to make inferences about causality, we interpret the output from this analysis as reflecting the potential size of a mediated effect were causal assumptions met.

Consistent with the approach recommended by Sterne *et al.* (39,40), and as is increasingly common practice in epidemiology, we avoided using an arbitrary cutoff to define significance and considered *p* values to reflect the strength of evidence for each finding. We therefore also avoided correction for multiple testing but interpreted the strength of evidence for the associations we observed in light of the number of tests, the findings from our sensitivity analyses, and the limitations in the study design.

### Multiple Imputation

To increase efficiency and minimize selection bias, we used multivariate imputation to impute trauma and confounder missing data up to the sample size with complete task and psychotic experience data (DTD, *n* = 3360; probability estimation, *n* = 3369). We used the ice command in STATA, carrying out 10 cycles of regression and creating 50 imputed datasets using 24 auxiliary variables related to outcomes and covariates and to missingness to make the missing-at-random assumption more plausible. Estimates in each imputed dataset were averaged following Rubin’s rules, accounting for uncertainty in the imputations and estimates (41). Analyses using imputed data are reported as the main results, with complete-case results presented in the Supplement.

## Results

### Participants

Compared with the excluded sample, the included participants were more likely to be female and have higher IQ and were less likely to be from a lower socioeconomic position, have childhood trauma, or have high genetic risk for schizophrenia (Table 1).

**Table 1:** Sample Characteristics of Participants Included in the Analytic Sample^a^^*,*^^b^

Participant Characteristic	Included (*n* = 3360), *n* (%)	Excluded (*n* = 10,819), *n* (%)	Odds Ratio (95% CI)	*p* Value
Female Sex	2101 (63%)	4805 (44%)	2.09 (1.92–2.26)	<.001
Low Income^c^	377 (13%)	1693 (24%)	0.48 (0.43–0.55)	<.001
Low Maternal Education^d^	516 (17%)	3207 (35%)	0.38 (0.34–0.42)	<.001
Low IQ^e^	350 (13%)	1075 (24%)	0.46 (0.40–0.52)	<.001
Family History of Mental Health Diagnoses	523 (17%)	1875 (19%)	0.85 (0.76–0.95)	.003
Childhood Trauma^e^	626 (21%)	2064 (28%)	0.70 (0.64–0.78)	<.001
High SCZ Genetic Risk^f^	415 (18%)	1087 (21%)	0.83 (0.73–0.94)	.004

DTD, draws-to-decision; SCZ, schizophrenia.

a Based on observed data on DTD task and psychotic experiences.

b The denominators vary for each measure due to missing data.

c Bottom quintile average household income at birth.

d <O-level.

e Childhood trauma reported 0–5 years of age.

f Top quintile; note that characteristics have been dichotomized for descriptive purposes only, with nondichotomized variables used in the analyses.

### Confounders and Belief Updating

Lower cognitive ability, female sex, and markers of lower socioeconomic status were, in the main, associated with more atypical belief updating (lower average DTD and greater disconfirmatory updating), reversal probability, and greater noise in the DTD and probability estimation tasks (Table 2). Increased genetic risk for schizophrenia was associated with greater reversal probability (*r*) and lower confidence in *r* in the probability estimation task.

**Table 2:** Distribution of Performance Parameters (Mean or %) in Relation to Confounders

Belief Updating Indices^a^	Female			Low Income^b^			Low Maternal Education^c^			Low IQ^d^			High SCZ PRS^e^		
	Yes	No	*p*	Yes	No	*p*	Yes	No	*p*	Yes	No	*p*	Yes	No	*p*
Average DTD	4.7	5.0	<.001	4.7	4.9	<.001	4.7	4.9	<.001	4.5	4.9	<.001	4.9	4.8	.129
Cost of Sampling	0.25	0.08	.002	0.25	0.17	.535	0.21	0.19	.582	0.11	0.21	.011	0.17	0.20	.321
Decision Noise	−0.05	−0.11	.164	0.03	−0.10	<.001	0.10	−0.11	<.001	0.28	−0.17	<.001	−0.06	−0.07	.565
Contrary Updating	1.81	1.60	<.001	1.9	1.7	<.001	1.97	1.7	<.001	2.38	1.55	<.001	1.81	1.71	.264
Reversal Probability	0.27	0.25	.001	0.28	0.26	.014	0.28	0.26	.008	0.31	0.25	<.001	0.28	0.26	.001
Adjustment Rate	0.65	0.65	.052	0.65	0.65	.204	0.65	0.65	.392	0.66	0.64	<.001	0.65	0.65	.780
Low Confidence^f^	45.7%	48.1%	.102	46.0%	46.7%	.727	47.3%	46.5%	.462	46.0%	46.8%	.513	49.6%	45.9%	.031
High Confidence^g^	31.8%	32.5%		31.8%	32.1%		33.6%	31.7%		32.8%	31.9%		28.1%	33.0%	
Inference Length	6.9	6.40	.017	6.8	6.7	.712	6.7	6.7	.715	6.7	6.7	.952	6.6	0.67	.821
Response Noise	−3.0	−2.89	<.001	−2.9	−3.0	<.001	−2.8	−3.0	<.001	−2.7	−3.1	<.001	−3.0	−3.0	.861

DTD, draws-to-decision; GCSE, General Certificate of Secondary Education; PRS, polygenic risk score; SCZ, schizophrenia.

a Average DTD and contrary updating are behavioral measures; all others are computational.

b Bottom quintile of average parental income.

c No GCSEs.

d Bottom quintile.

e Top quintile.

f Bottom tertile vs. middle.

g Top tertile vs. middle. Sample based on imputed estimates. *p* values based on analyses using continuous/ordinal measures of confounders where available rather than binary ones. Note that characteristics have been dichotomized for descriptive purposes only, with nondichotomized variables used in the analyses.

### DTD Task and Psychotic Experiences

Overall, 125 participants (3.9%) had past-year frequent or distressing psychotic experiences at age 24. Individuals with greater average DTD had a reduced odds of psychotic experiences, although there was little evidence of this association after adjusting for confounding (OR_adj_ = 0.93, 95% CI = 0.84 to 1.04, *p* = .226) (Table 3).

**Table 3:** Belief-Updating Parameters and Frequent or Distressing PEs at Age 24 Years^a^

Belief Updating Indices^b^	Unadjusted		Adjusted^c^	
	OR (95% CI)	*p*	OR (95% CI)	*p*
DTD Task				
Average DTD	0.89 (0.80–0.99)	.031	0.93 (0.84–1.04)	.226
Cost of sampling	0.95 (0.86–1.06)	.384	0.96 (0.86–1.07)	.467
Decision noise—linear	1.11 (0.99–1.24)	<.001	1.05 (0.94–1.18)	.006
Decision noise—quadratic	1.09 (1.04–1.14)		1.08 (1.03–1.14)	
Probability Estimation Task				
Contrary updating	1.08 (0.99–1.18)	.075	1.04 (0.94–1.15)	.435
Reversal probability	1.99 (0.78–5.07)	.150	1.62 (0.61–4.32)	.336
Adjustment rate	1.43 (0.16–12.88)	.751	1.05 (0.11–9.72)	.965
Low confidence	1.13 (0.71–1.78)	.610	1.11 (0.70–1.77)	.657
High confidence	1.16 (0.72–1.89)	.538	1.13 (0.69–1.85)	.618
Inference length	1.03 (0.93–1.14)	.565	1.02 (0.92–1.14)	.663
Response noise	1.23 (1.04–1.45)	.015	1.12 (0.94–1.33)	.192

DTD, draws-to-decision; PE, psychotic experience.

a Imputed sample (*n* = 3360 for the DTD task, *n* = 3244 for response noise, *n* = 3369 for rest of the probability estimation task).

b Average DTD and contrary updating are behavioral measures; all others are computational.

c Adjusted for working memory, IQ, executive functioning, sex, social class, crowded living conditions, income, trauma, and genetic risk for schizophrenia.

**Figure 1:**
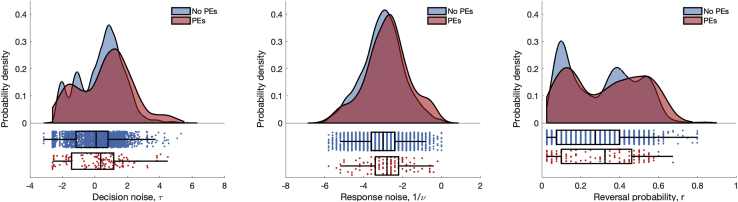
Probability density distributions (top panels) and individual counts with boxplot (bottom panels) of the parameter estimates for decision noise (in the draws-to-decision task) and response noise and reversal probability (in the probability estimation task) shown for participants with (red) and without (blue) psychotic experiences (PEs). The evidence for an association between these parameters and PEs was robust only for decision noise. There was no evidence for an association with response noise after adjusting, and reversal probability showed an association only when treated as a binary measure in the sensitivity analyses.

Results from the computational model, however, indicated that those with greater decision noise had an increased likelihood of frequent or distressing psychotic experiences (*p* < .001) (Figure 1), and this persisted after adjusting for confounders (*p*_adj_ = .006). Adjusting for IQ, income, maternal education, and trauma had the strongest effect on attenuating this and other associations with psychotic outcomes. Cost of sampling was not associated with psychotic experiences.

### Probability Estimation Task and Psychotic Experiences

There was no evidence to support an association between updating to disconfirmatory evidence and psychotic experiences (OR_adj_ = 1.04, 95% CI = 0.94 to 1.15, *p* = .435) (Table 3).

When examining the parameters derived using computational modeling, those with greater response noise were more likely to have psychotic experiences (OR = 1.23, 95% CI = 1.04 to 1.45, *p* = .015) (Figure 1), but there was no evidence of association after adjusting for confounders (OR_adj_ = 1.12, 95% CI = 0.94 to 1.33, *p* = .192). None of the other computational parameters were associated with psychotic experiences.

### Exposure to Trauma and Abnormal Belief Updating Processes

**Figure 2:**
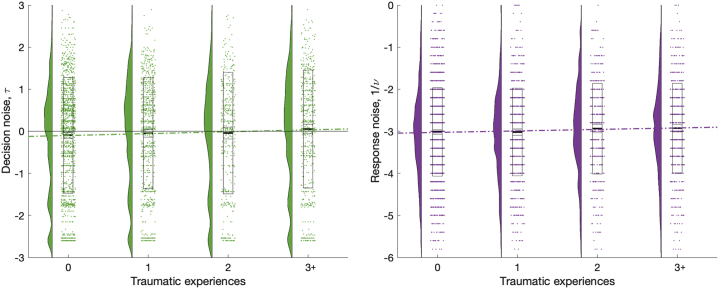
Probability density distribution (left panels) and individual counts with boxplot (right panels) of the parameter estimates for decision noise (in the draws-to-decision task) and response noise (in the probability estimation task) shown for each level of trauma coded in terms of the number of different trauma types experienced (from none to three or more). There was robust evidence for an association of both parameters with trauma.

In total, 66.5% of the imputed sample reported exposure to at least one type of trauma, and 23.7% were exposed to three or more types of trauma between 0 and 17 years of age. There was strong evidence of associations between trauma and a lower average DTD (Beta_adj_ = −0.07, 95% CI = −0.12 to −0.02, *p* = .007) (Table 4), greater decision noise in the DTD task (OR_adj_ = 1.16, 95% CI = 1.04 to 1.29, *p* = .007) (Figure 2), and greater response noise in the probability estimation task (Beta_adj_ = 0.05, 95% CI = 0.02 to 0.08, *p* = .005) (Figure 2). There was no evidence of association between trauma and the other computational parameters.

**Table 4:** Association Between Exposure to Trauma and Belief Updating Parameters^a^

Belief Updating Parameters^b^	Effect	Unadjusted		Adjusted^c^	
		Effect Size (95% CI)	*p* Value	Effect Size (95% CI)	*p* Value
DTD Task					
DTD	β	−0.08 (−0.13 to −0.02)	.003	−0.07 (−0.12 to −0.02)	.007
High cost of sampling	OR	1.05 (0.94 to 1.16)	.386	1.05 (0.95 to 1.17)	.333
High decision noise	OR	1.19 (1.07 to 1.32)	.001	1.16 (1.04 to 1.29)	.007
Probability Estimation Task					
Contrary updating	β	0.02 (−0.00 to 0.04)	.068	0.01 (−0.01 to 0.03)	.320
High reversal probability	OR	1.03 (0.92 to 1.15)	.584	1.01 (0.90 to 1.13)	.895
Adjustment rate	β	0.0 (−0.00 to 0.01)	.102	0.00 (−0.00 to 0.01)	.170
Low confidence	RRR	1.05 (0.97 to 1.15)	.213	1.04 (0.96 to 1.14)	.318
High confidence	RRR	1.06 (0.97 to 1.16)	.180	1.06 (0.97 to 1.16)	.230
Inference length	OR	0.98 (0.92 to 1.04)	.511	0.98 (0.92 to 1.04)	.513
Response noise	β	0.07 (0.03 to 0.10)	<.001	0.05 (0.02 to 0.08)	.005

DTD, draws-to-decision; OR, odds ratio; RRR, relative risk ratio.

a Imputed sample (*n* = 3429 for the DTD task, *n* = 3311 for response noise, *n* = 3438 for rest of the probability estimation task).

b Average DTD and contrary updating are behavioral measures; all others are computational.

c Adjusted for sex, income, crowding, social class, maternal education, and genetic risk for schizophrenia.

### Mediation Analysis

Because decision noise in the DTD task was associated with both trauma and psychotic experiences, we examined the extent to which this mediated the association between trauma and psychotic experiences. Exposure to three or more types of trauma was associated with a 3.6-fold increase in odds of psychotic experiences at age 24 (OR_adj_; 95% CI = 2.43 to 5.58). However, there was little evidence to suggest that the association between exposure to trauma and psychotic experiences was mediated by decision noise on the DTD task (natural indirect effect: OR_adj_ = 1.03, 95% CI = 0.99 to 1.07, percent mediated 2.2%) (Table S2).

### Psychotic Disorder

A total of 71 participants (2.1%) met criteria for a psychotic disorder or at-risk mental state at age 24. There was weak evidence of association with average DTD (OR_adj_ = 0.86, 95% CI = 0.74 to 1.00, *p* = .057), while the association with DTD decision noise (*p*_adj_ = .008) was strong (Table S3). There was strong evidence of association between trauma and psychotic disorder (OR_adj_ = 3.62, 95% CI = 2.10 to 6.22, *p* < .001), although DTD decision noise mediated only a small proportion of this effect (natural indirect effect: OR_adj_ = 1.03, 95% CI = 0.98 to 1.08, percent mediated 2.5%).

### Psychopathology Symptom Specificity

Overall, 59 participants (1.7%) had delusions, 89 (2.6%) had hallucinations (20 [0.6%] reported both symptoms), 246 (7.2%) had moderate to severe depressive disorder, and 325 (9.5%) had an anxiety disorder. There was little evidence that any of the belief updating measures were more strongly associated with delusions than hallucinations or vice versa (Table S4). There was weak evidence that individuals with an anxiety disorder had a higher average DTD than those with psychotic experiences but no evidence that other parameters were associated with depression or anxiety or to support specific associations of belief updating with psychotic experiences (Table S5).

### Sensitivity Analysis

Results using different cutoffs (85th and 95th percentiles) for binary measures of cost of sampling, decision noise, and reversal probability as outcomes showed a slightly different pattern of results. Results using collapsed binary measures for these variables as exposures showed weaker evidence of associations between DTD decision noise and psychotic experiences but stronger evidence for reversal probability and psychotic outcomes (strongest evidence at 85th percentile: OR_adj_ for psychotic experiences = 1.74, 95% CI = 1.12 to 2.69, *p* = .013; OR_adj_ for psychotic disorder = 2.30, 95% CI = 1.32 to 4.04, *p* = .004) (Figure 1; Tables S6 and S7). Results were substantively unchanged using an ordinal measure of number of psychotic experiences as the outcome (Table S8).

### Complete-Case Analysis

Overall, estimates for the DTD task using complete-case data were similar to those using imputed data, whereas estimates for the probability estimation task parameters differed more substantially. All estimates were less precise, and evidence of association between parameters and psychotic experiences was weaker (Table S9).

### Parameter Recovery Analysis

We simulated data using typical ranges of parameter values and tested whether model inversion could accurately recover the parameters of the winning model. Both parameters associated with psychotic experiences or trauma—reversal probability and response noise—were estimated very reliably (both *ρ* > .9) (detailed in Supplemental Results).

## Discussion

In this study, we used computational modeling to characterize the mechanisms underlying belief updating in a large population-based sample and examined their association with trauma and psychotic experiences. Psychotic experiences were associated with decision noise—a computational model parameter—in the DTD task. In the sensitivity analyses, we found weak evidence of an association with a higher reversal probability—another computational model parameter—in the probability estimation task. However, there was no convincing evidence that the typically used behavioral measures were associated with psychotic outcomes, suggesting that if anything, computational modeling can more reliably detect abnormal cognitive processes. IQ and sociodemographic characteristics showed strong evidence of association with belief updating measures, and most estimates were substantially attenuated after adjusting for these factors. This finding supports the notion that our behavioral and computational measures pick up on general, noninferential factors rather than specific belief updating mechanisms (42). Trauma exposure was associated with psychotic experiences and increased noise parameters in both belief updating tasks, but almost none of trauma’s effect on psychotic experiences was mediated by the latter. Together, these findings challenge the hypothesis that atypical belief updating mechanisms, as indexed by the computational models we used and, even more so, by typically used behavioral measures, underlie the development of psychotic phenomena. The results also indicate that belief updating (as indexed by the computational models and behavioral measures we used) is unlikely to be a mediating process by which trauma leads to psychosis.

### Abnormal Belief Updating and Psychotic Experiences

Evidence of association between decision noise in the DTD task and psychotic experiences was strong. However, while increased noise parameters in this and other tasks have also been found to be associated with schizophrenia (23) [although not always (25)], it is unclear how informative this computational index is with respect to specific mechanisms. In particular, this finding could mean that cognitive processes are more stochastic in those with (or at risk of) psychosis but, alternatively, might reflect underlying mechanisms that were not modeled.

Subjective reversal probability—i.e., expectation of change—was associated with frequent or distressing psychotic experiences and with clinical disorder in this population sample but only in sensitivity analyses when examining the effects of the highest scorers on this measure. Hence, the strength of evidence for an association between this parameter and psychotic outcomes in our study has to be regarded as weak, particularly in the context of the number of analyses undertaken. The lack of a relationship between the continuous measure of reversal probability and psychotic outcomes and the bimodality of its distribution (Figure 1 and Figure S3) may indicate that the higher mode alone is associated with psychosis risk. Genetic risk for schizophrenia was also associated with a higher reversal probability—and also greater uncertainty about this prior belief—but did not explain the association between reversal probability and psychotic experiences.

If taken at face value, an increased expectation of change probably describes more unstable beliefs rather than a specific expectation that contingencies are more likely to change, although we cannot discriminate between these possibilities here. This relationship of increased expectation of change with psychotic experiences is consistent with findings of increased belief instability in people with schizophrenia (20), including unmedicated subjects (43), and in nonclinically ascertained individuals with delusional ideation (24). However, it is not consistent with the increased adjustment rate that a uniform overweighting evidence hypothesis would predict (22), because in this case, confirmatory and disconfirmatory evidence would be overweighted by the same amount. In situational judgments [e.g., ranking the plausibility of different accounts for a social scenario (18)] rather than probabilistic ones (e.g., predicting the outcome of an event under conditions of uncertainty), people with delusions are less likely to revise beliefs in light of contradictory information (bias against disconfirmatory evidence) (19,44). At present, the existing evidence cannot reconcile these seemingly contradictory findings.

Overall, however, probably the most striking aspect of our findings is the fact that the evidence for an association between any specific belief updating mechanism and psychotic experiences was, at best, weak. Considering the large sample size of our study, our finding might therefore suggest that previous claims regarding the importance of atypical belief updating mechanisms (as measured by the two tasks we used) for the emergence of psychotic experiences have to be treated with caution. However, it is, of course, possible that psychotic experiences and atypical belief updating show strong associations only in a specific clinical subgroup of patients or a specific illness stage, a question that we cannot address with our data.

Some studies have suggested that an atypical number of draws in the DTD task is specifically associated with delusions (13,45) and that altered inference processes may relate differently to hallucinations and delusions in other tasks (46,47). In our study, we found little evidence to support any difference between hallucinations and delusions. Similarly, while abnormal belief updating mechanisms have been associated with mood disorders in one study (20), we found no associations with depression or anxiety, except perhaps for a tendency for individuals with anxiety disorder to request more beads in the DTD task. However, there was little evidence to support the presence of disorder-specific belief updating mechanisms in our study. One difference between previous studies using the DTD task and our study is noteworthy: previous studies typically used only one sequence of beads for all participants. We presented five sequences per participant, and these were randomly sampled from 16 possible sequences. This approach increases the generalizability of the data (see the Supplement for more details).

### Trauma, Psychotic Experiences, and Abnormal Belief Updating

The dose-response association between trauma exposure and greater decision and response noise in the DTD and probability estimation tasks suggests that trauma results in greater cognitive stochasticity as opposed to more specific changes in belief updating (although we cannot rule out additional changes in specific cognitive strategies not modeled in our tasks). However, while DTD task decision noise was associated with both childhood trauma and psychotic experiences, it did not mediate the trauma–psychotic experiences association. Our data are cross-sectional, but nevertheless they suggest that even if decision stochasticity occurred prior to the onset of psychotic experiences, only a very small proportion of the association between trauma and psychotic experiences would be explained by this measure. This finding challenges the idea that trauma-induced changes in belief updating are an important mechanism contributing to psychosis in contrast to the evidence around psychological mechanisms (48), although other belief updating processes not modeled here might be important.

### Strengths and Limitations

Strengths of our study include the use of comprehensive measures of trauma and semistructured interviews for psychotic outcomes to minimize measurement error and inclusion of a broad range of prospectively assessed confounders. A further strength is our use of computational modeling to investigate mechanisms underlying belief updating, with results showing stronger effects for computational parameters than behavioral ones and with effect estimates generally being less affected by confounding than for behavioral measures.

However, our results need to be interpreted in the context of several limitations. First, it remains possible that residual confounding exists, as is the case for all observational study designs, particularly genetic confounding. Second, the cross-sectional nature of the belief updating and psychotic experience data precludes us from making inferences about whether abnormal belief updating has a causal effect on psychotic experiences, although we can be confident that little of the association between trauma exposure and subsequent psychotic experiences is mediated through DTD task decision noise. Third, as with most cohort studies, there was substantial attrition over time. We used multiple imputation using a range of auxiliary variables to make the missing-at-random assumption more plausible, but it is nevertheless possible that selection bias remained. Fourth, our findings for decision noise and particularly for reversal probability need to be interpreted in the context of the number of analyses undertaken, and replication in other samples is required. For instance, the DTD task involved testing for one noncomputational and two computational parameters, and the probability estimation task involved one noncomputational and five computational parameters. Fifth, the sparse data generated by the DTD task provided limited potential for computational modeling, and it is possible that these analyses missed other belief updating strategies. Finally, while we were able to identify individuals meeting criteria for a psychotic disorder, approximately 50% had not sought help for their symptoms, and thus, cases in our sample are likely to be less severe than those recruited through clinical services; hence, our findings might not be fully comparable to previous studies.

### Implications and Conclusions

Our work shows that it is possible to collect data amenable to computational modeling at scale. The findings provide some evidence that computational parameters may relate more directly to underlying mechanisms compared with behavioral measures. In the context of previous claims and given the large sample size of our study, however, the most striking finding is the weakness of the evidence linking computational and behavioral indices of specific belief updating mechanisms to psychotic experiences and psychotic disorder. The most robust finding for an association with psychotic experiences was found for decision noise, a potentially unspecific index for the stochasticity of cognitive processes or, alternatively, for unmodeled factors (20,22). Decision noise also related to past exposure to trauma but did not mediate the relationship between trauma and psychotic experiences. Further longitudinal studies are required to understand how trauma leads to psychosis and to examine whether atypical belief updating processes play a role in the etiology of psychosis.
