# Supplementary material for: A Computational Analysis of Abnormal Belief Updating Processes and Their Association With Psychotic Experiences and Childhood Trauma in a UK Birth Cohort
Source: Biol Psychiatry Cogn Neurosci Neuroimaging. 2022 Jul;7(7):725–34. doi: 10.1016/j.bpsc.2021.12.007 (PMC9259502; doi:10.1016/j.bpsc.2021.12.007)
Supplement: Supplementary Material [file mmc1.pdf]

A Computational Analysis of Abnormal Belief-Updating Processes and Their Association With Psychotic Experiences and Childhood Trauma in a UK Birth Cohort

Supplementary Information

Contents

Supplementary Methodology Information 3

Further information on assessment of trauma 3

Further information on Assessment of Psychotic Outcomes 3

Behavioural Tasks: Procedure and Design 5

Draws-to-Decisions Task 5

Probability Estimation Task 7

Draws to Decision Task Parameters 8

Cost of sampling 8

Decision Noise 10

Probability Estimation Task Parameters 10

Reversal probability, *r*: 17

Adjustment rate, *a*: 17

Window length, *L*: 17

Confidence in *r* (Dirichlet parameter), $\Pi(r)$: 17

Response noise, *1/ v*: 17

Confounding Variables 18

Supplementary Results 18

Missing data 20

Figure S1: Flow chart of participant inclusion 21

Figure S2: Model comparison for Probability Estimation task models 22

Figure S3: Distribution of belief-updating indices and derivation of categorical and binary measures 23

Figure S4: Simulations showing the effects of increasing *r* on inference in the Probability Estimates task 24

Table S1: Correlation between belief-updating behavioural measures and computational parameters 26

Table S2: Mediation analysis of trauma (exposure to 3+ trauma types), higher decision noise (draws to decision task) and PEs at age 24 years 26

Table S3: Belief-updating indices and psychotic disorder at age 24 years[1] 27

Table S4: Bivariate analysis of belief-updating indices, hallucinations and delusions[1] 28

Table S5: Multivariable analysis of belief-updating indices and depression, anxiety and psychotic experiences 29

Table S6: Sensitivity analyses of belief updating and psychotic outcomes using percentile cut-offs for non-normal continuous measures[1] 30

Table S7: Sensitivity analyses of trauma and belief updating using different percentile cut-offs for non-normal continuous variables[1] 31

Table S8: Belief-updating parameters and ordinal measure of number of frequent or distressing PEs at age 24 years[1] 32

Table S9: Belief-updating indices and frequent or distressing PEs at age 24 years in the past 12 months in complete-case sample[1] 34

Table S10: Association between exposure to trauma and belief-updating parameters (complete-case sample) 35

Supplementary References 36

Supplementary Methodology Information

Further information on assessment of trauma

A binary measure of trauma was derived from 121 questions selected from 48 assessments completed contemporaneously by the child or their parents from the ages of 0-17 years, and from a questionnaire completed by the young person at age 22 to supplement information on sexual abuse that was almost entirely parent-reported in earlier questionnaires. The questions covered sexual, physical and emotional abuse, as well as emotional neglect, bullying and exposure to domestic violence, and were selected on the basis that they would be deemed as being highly upsetting for almost everyone who experienced them. Participants were coded as having been exposed to a trauma if they endorsed any of the questions relating to these traumas between ages 0 to 17 years. Participants were coded as non-exposed if they had not endorsed any of the questions, and participated in at least 50% of assessments. For further details on how this measure was derived see Croft et al 2019[1].

Further information on Assessment of Psychotic Outcomes

The interviewers were psychology graduates trained in using the PLIKSi, and blind to previous PLIKS assessments. Interviewers had to score >0.9 agreement with 'gold-standard' ratings on 2 audio-recorded interviews before they were able to start collecting data for the study. At regular intervals, a psychiatrist rated samples of recorded interviews to ensure that the interviewers were rating experiences correctly.

At-risk mental state for psychosis

Individuals with a current at-risk mental state for psychosis were identified by relating the PLIKS interview data at age 24 to the Structured Interview for Prodromal Symptoms (SIPS)[2]

definitions of prodromal symptoms and the Comprehensive Assessment of At-Risk Mental State (CAARMS)[3] criteria (see Sullivan et al 2020[4] for more detail).

Psychotic disorder

We classified individuals as having a psychotic disorder if i) they were rated as having a definite psychotic experience not attributable to the effects of sleep or fever, ii) this had recurred regularly (at least once per month) averaged over the previous 6 months, and iii) they reported this as either very distressing, or having a very negative impact on their social or occupational functioning, or having led them to seek help from a professional source[4].

Behavioural Tasks: Procedure and Design

Each participant took part in two separate tasks: a 'Draws-To-Decision' (DTD) task and a 'Probability Estimation Task'. Both tasks have been used to assess belief formation in clinical populations in a substantial number of previous studies (see main text for details). For both tasks, each participant was instructed in person by a trained and experienced experimenter, who answered the participants' questions, carefully checked task comprehension, and conducted a practice with the participant to ensure they understood the instructions. To further support the experimenter's judgment, future studies might benefit from a more formal assessment of task comprehension at the end of the experiment. Verbal instructions were supported by on-screen text and illustrations (detailed in the next two sections). Participants received no re-imbursement other than travel expenses to attend the clinic.

Draws-to-Decisions Task

At the start of the experiment, the experimenter presented participants with an illustration of two jars with two different colours of beads. Participants were told that the jars contain 100 beads with inverse proportions of coloured beads at a ratio of 80:20. They were then told that the computer would randomly choose a bead from one of the jars, show it to them, and then put it back in the jar. After each presentation of a bead, the participant could either state which jar the bead was drawn from or request to see another bead, which was drawn from the same jar. There was no time limit to making a decision. Participants could request up to ten beads before deciding from which jar the beads were being drawn. The number of beads that were requested is referred to as 'draws'.

The Draws-to-Decision task was completed five times with five different sequences of beads, and the participants were told that the computer selects the jar at random each time. Most previous studies that used the DTD task employed only one sequence for all participants. This design choice assumes that the stimulus is a 'fixed effect'[5], which increases the probability that idiosyncratic aspects of the sequence have unintended effects on participants' responses that will not generalise to other sequences. As a more robust strategy, we chose to vary the sequences, present more than one sequence per participant, and present different sequences to different participants. The sequences of beads that were randomly chosen from and presented in five trials to each participant were as follows (0 = Red; 1 = Blue):

0,0,0,0,1,0,0,0,0,0
1,0,1,1,1,1,0,1,1,1
1,0,0,0,0,0,0,0,0,0
1,0,0,1,1,1,1,1,1,0
1,0,0,1,1,0,0,0,0,0
1,1,0,1,0,1,1,1,1,1
0,0,0,1,1,0,0,1,0,0
1,1,0,1,1,1,1,1,1,1
1,1,1,1,0,1,1,1,1,1
0,1,0,0,0,0,1,0,0,0

0,1,1,1,1,1,1,1,1,1
0,1,1,0,0,0,0,0,0,1
0,1,1,0,0,1,1,1,1,1
0,0,1,0,1,0,0,0,0,0
1,1,1,0,0,1,1,0,1,1
0,0,1,0,0,0,0,0,0,0

The on-screen text and illustrations that supported the experimenter's verbal task instructions were as follows:

There are 2 jars filled with beads – each jar has 100 beads

The red jar is mostly red with some blue, the blue jar is mostly blue with some red

You cannot see these jars

The computer will pick beads from one of these jars

You have to decide which jar the beads are coming from

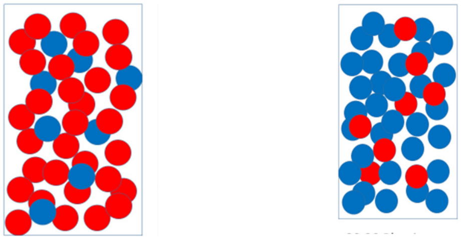

Red jar Blue jar

80 Red **80 Blue**

20 Blue **20 Red**

The computer will randomly choose a bead from one of the jars, show it to you, and then put it back in the jar

You can ask the computer to show you more beads to help you decide which jar the beads are coming from, up to 10 beads in total

You should decide as soon as you are sure about which jar the beads are coming from

You will see the previous beads you have seen on the bottom of the screen

Current bead

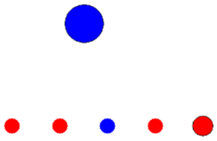

First bead Previous bead

You will do this task 5 times - each time, the computer will choose a jar at random

Probability Estimation Task

The basic setup of the Probability Estimation Task was identical to that of the previous task: participants were presented with an illustration of two jars, again filled with two different colours of beads. They were told that the jars contain 100 beads with inverse proportions of coloured beads at a ratio of 80:20. Again, they were informed that the computer would randomly choose a bead from one of the jars, show it to them, and then put it back in the jar. Participants were told that they would be shown a sequence of 30 beads. Every time a bead was presented, the participant had to rate how certain they were about which jar the beads were being drawn from. Every participant was shown the entire sequence of 30 beads. They were also told that the jar from which the beads were being drawn may or may not change during the task at any point in the sequence and may change multiple times. Participants were not able to see the sequence of previously presented beads in this task. Similar to the previous task, there was no time limit to making a decision.

The bead sequence for the Probability Estimation Task was identical for each participant (0 = Red; 1 = Blue):

111 0 1 0 1111111 0 1 0000 1 000000 1 000

The on-screen text and illustrations that supported the experimenter's verbal task instructions were as follows:

The computer will randomly choose a bead from one of the jars, show it to you, and then put it back in the jar

This time you will be shown more than 10 beads

Every time you are shown a bead you have to rate how sure you are of which jar the beads are coming from

Use the mouse to click along the scale

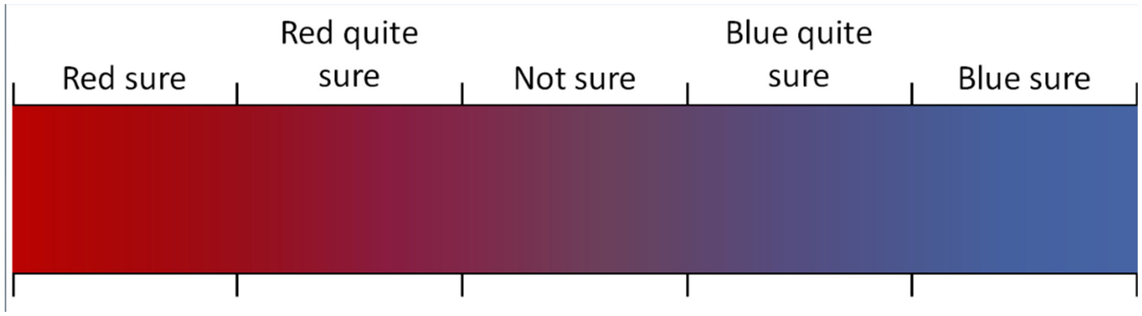

Draws to Decision Task Parameters

Two behavioural measures of the task were derived. First, Draws to Decision (DTD), as widely used in previous literature[6], indexes the average number of beads drawn by participants across five trials of the task. Second, the 'Jumping to Conclusions' bias that is also widely used in previous literature[7,8] is a binary measure that indexes whether a decision was reached based on an average of 2 or fewer beads across the trials.

Two computational parameters were derived using a fully Bayesian model, as described in previous studies[9,10]. The task consists of a partially-observable Markov decision process. It is a decision process in the sense that participants only have to decide when they have enough evidence to declare a decision in favour of one of the jars or the other. It is Markovian, because at every point in the task, after every draw, the evidence can be simply summarized as the number of draws of each colour drawn. Given this 'state description', it does not matter what the past history of draws is. Finally, it is partially observable because the coloured draws induce uncertain beliefs about the underlying jars, which are not directly observed. The task is so simple that a full dynamic programming solution can be computed. This means, all the possible draw scenarios between the current draw and the end of each trial can be considered, and on their basis the value of declaring for one colour or the other, or drawing again, can be calculated. Although this is easy for the computer model, it is hard for people to do. Fortunately, we can parameterize the 'effective depth' of people's cognition, which then becomes a measure of goal-directed or future-thinking ability, by a decision or cognitive noise parameter. This 'blurs' the model's ability to consider deep scenarios, effectively discounting cognitively distant potential observations. This is the decision-noise parameter.

Cost of sampling

As the value (i.e. penalty or reward) for drawing additional beads is not stated, the cost of sampling estimates the subjective value that participants attribute to requesting additional beads. A high cost of sampling could account for the JTC bias by demonstrating that there is a consistent strategy where a greater cost is the basis for requesting less information before deciding. A higher perceived cost of sampling would indicate a greater desire to complete the task quickly, which may be due to motivational factors including subjective opportunity cost (i.e, one has better things to do with one's time than this task!), or possibly intolerance of uncertainty or subjective cost to self-esteem when requesting further information[9,10]. The cost of sampling index was not normally distributed and difficult to transform; therefore, when examining this as an outcome, we derived a dichotomous variable which grouped the top 10% of participants versus the bottom 90% (Figure S3). As sensitivity analyses we also examined cut-offs at the 85th and 95th percentiles. When modelled as an exposure, we used the continuous measure, with/without a quadratic term, and also examined variables dichotomised at different cut-offs in sensitivity analyses.

The model is described in detail elsewhere[9,10] but in brief, these parameters affect decision making in the following way. Cost of sampling C^S is defined relative to the (fixed) cost of making the wrong decision C^W; the possible decisions being that the jar is blue (D_B), the jar

is red (D_R), or to sample again (D_S). The value Q of sampling again and getting a red (r) or blue (b) bead, given the number of beads drawn n_d of which n_r were red, and given the underlying jar is red (R) rather than blue (B), is computed as:

$$Q(D_S; n_d, n_r) = C^S + C^W \cdot P(R|n_d, n_r) +$$
$$P(R|n_d, n_r) \cdot [V(n_d + 1, n_r + 1)P(r|R) + V(n_d + 1, n_r)P(b|R)] +$$
$$P(B|n_d, n_r) \cdot [V(n_d + 1, n_r + 1)P(r|B) + V(n_d + 1, n_r)P(b|B)]$$

Here the probabilities are computed using Bayes theorem and the values of states V are the summed products of values of actions that can be taken from those states and the probabilities of taking those actions. The probability of taking action a (rather than b) in state $s = (n_d, n_r)$ is computed according to a standard softmax function, incorporating decision noise τ:

$$P(a|s) = \exp\left(\frac{Q(a,s)}{\tau}\right) \Big/ \sum_{b\in\{D_R,D_B,D_S\}} \exp\left(\frac{Q(b,s)}{\tau}\right)$$

Values of states far in the future therefore depend on multiple actions at successive timepoints, and thus as τ becomes higher, the effective planning horizon becomes shorter, as it becomes less likely that sequences of optimal actions will be selected and so values of distant actions have little effect on values of current states.

We did not fit multiple models to the data and perform model selection for several reasons. The first is that this model and its variants have been validated for this task in multiple studies using both clinical and community samples[9,11,12]. Second, it is already very compact, estimating just two parameters, which is an advantage when so little data (five trials) is available per participant. Third, and related to this, the compactness of the model allowed us to easily use model-fitting techniques that are likely to optimise detection of individual variability, i.e. correlation with unmodelled participant characteristics as we shall see in the next section. Fourth, this model has the ability to capture a number of heuristic strategies that participants use (such as collecting all the data available and then deciding, as described above) which is important in large samples where it is unlikely that all participants will use the same cognitive strategy. Finally, our aim here was to test hypotheses closely related to the constructs captured by this model.

Model-fitting

The model-fitting approach used here relied on a full mapping of the likelihood function over a fine parameter grid covering the entire range of psychological interest of the two parameters in question. The nature of data collected in large, multi-measure studies like the present one poses particular challenges. The often-used maximum-likelihood parameter estimates are often very noisy due to the small number of datapoints per participant, necessitating the use of prior distributions over parameters. We have shown that using empirical prior distributions which do not take into account the variables external to the

model (such as genetic, demographic and psychometric scores) is prone to suppressing variability due to these external variables of interest, making their effect harder to detect[13]. This theoretical work suggested that when no exploratory analyses are to be performed, the most sensitive approach is to incorporate hypothesis testing in the construction of the empirical priors; however, a compromise which is almost as good and allows greater flexibility for exploratory analyses is to use weak, regularizing priors. This is the approach we used here, diverging from the hierarchical (mixed-effects) fits of previous work which dealt with a priori defined participant groups[9,10]. Here, we constructed 200 x 200 parameter grids in log-temperature x log-subjective-cost space. These spanned the range from almost-deterministic to completely random behaviour (log decision noise from -5 to 5) and subjective cost of sampling (log (-cost) from -5 to 50 which was negligible compared to the reference cost of getting the answer wrong, to cost high enough to result in immediately declaring a decision, at the very first, obligatory item of information seen). Along each parameter dimension, we imposed a weak Gaussian prior of mean 0.83 and SD of 2.86. The mean was based on preliminary analysis of a random 100 participants by simple maximum-likelihood, and the SD was taken to be such that +/-3 SD was approximately twice as broad as the grid range. We then calculated the posterior probability of the data at each point of the grid, approximating the maximum with a parabolic fit.

Decision Noise

Decision noise in this task is usually not normally distributed and difficult to transform. As described in 'Methods', participants are simply told to gather information until they want to declare their decision, but no external cost was imposed per item of information in our simple version of the task. Therefore, sophisticated participants who are patient can follow a simple strategy, which is to gather all the information available and then decide (rather than consider step-wise decisions). The model can capture this well, as participants with very low decision noise, resulting in a bimodal distribution. Similarly, the model can capture very erratic participants as having very high decision noise. This flexibility results in a psychologically meaningful, but highly non-normal distribution. For analysis of this as an outcome, we thus grouped the top 10% of participants versus the bottom 90%, though as sensitivity analyses we also examined cut-offs at the 85th and 95th percentiles. When this was an exposure, we modelled the continuous measure, with/without a quadratic term, and also examined variables dichotomised at different cut-offs in sensitivity analyses.

Probability Estimation Task Parameters

A behavioural measure of 'disconfirmatory updating' was derived, based on previous studies of the task[14,15]. This measure is the absolute value of the change in estimation after seeing a bead of a different colour to at least two identically-coloured preceding beads (e.g. the change in estimation when seeing a blue bead after seeing two or more red beads). This type of update happened several times during the sequence, so each participant's disconfirmatory update score was the mean of all these updates.

For computational modelling of the probability estimation task, we tested five models and selected the winning model based according to the Bayesian Information Criterion (BIC). The models were:

1) Pearce-Hall model

The Pearce-Hall model[16] is similar to a Rescorla-Wagner model – i.e. it learns contingencies by incremental updating by the product of a prediction error and a learning rate – but its learning rate is not fixed, but varies from trial to trial: the learning rate takes the value of the prediction error on the previous trial, unless this was 0, in which case the learning rate remains the same as it was on the previous trial. We used the Pearce-Hall model implemented in the Tapas Hierarchical Gaussian Filter toolbox (version 5), available from http://www.translationalneuromodeling.org/tapas/: this analysis used the perceptual model (which incrementally updates beliefs about the jars) 'tapas_ph_binary' and the response model (which maps from beliefs to the participant's response on the sliding scale) 'tapas_beta_obs', and the standard (weakly informative) prior settings over parameters. These were: initial $p(jar = R) \sim N(0.5,0)$, learning rate $\alpha \sim N(0.5,1)$, stimulus intensity $S \sim N(0.1,8)$ and response stochasticity determined by the precision of a beta distribution $\nu \sim N(128,4)$ (where $\nu := \alpha + \beta$, the conventional parameters of the beta distribution). The variances given here refer not to the parameters' native space, which in many cases is bounded, but to the unbounded space they were transformed to for estimation purposes.

2) Hierarchical Gaussian Filter: 2 levels

The HGF is a hierarchical Bayesian inference scheme which gives a principled account of how beliefs are updated on acquiring new data, using individual priors over parameters, and has been used many times(e.g[14,17]) model this or similar tasks. Please see these references for full descriptions of the model: the following is a summary. This analysis used the perceptual model 'tapas_hgf_binary_scaled' and response model 'tapas_beta_obs'.

Bayes' theorem allows us to calculate the posterior belief that either jar is currently the source of the beads, if we combine the likelihoods that characterise each jar with a prior belief about the probability of either jar being correct. The likelihoods characterising each jar were simply the proportions of bead colours within each jar. The difference between prior and posterior belief constitutes a prediction error that can be used to learn the higher-order dynamics of changes between jars. In other words, the prediction errors that occur in the face of Bayes-optimal predictions are used to infer the current state of the environment.

At the bottom of the model is the bead drawn $u^{(k)}$ on trial k and the probability $x_1^{(k)}$ that draws are coming from the red jar. At the level above this is x_2, the tendency towards the red jar. For $x_2 = 0$, both jars are equally probable. This quantity is hidden from the participant and must be inferred: the participant's posterior estimate of x_2 is μ_2, and the participant's posterior estimate of the probability of the jar being red is a sigmoid function of this quantity, $s(\mu_2)$ – equivalent to the prediction (denoted by ^) on the next trial $\hat{\mu}_1^{(k+1)}$. .

Before seeing any new input on trial k the model's expected jar probability $\hat{\mu}_1^{(k)}$ and precisions (inverse variances) $\hat{\pi}_1^{(k)}, \hat{\pi}_2^{(k)}$ of the expectations at each level are given by:

$$\hat{\mu}_1^{(k)} \equiv s\left(\mu_2^{(k-1)}\right)$$

$$\hat{\pi}_1^{(k)} \equiv \frac{1}{\hat{\mu}_1^{(k)}(1-\hat{\mu}_1^{(k)})}$$

$$\hat{\pi}_2^{(k)} \equiv \frac{1}{\sigma_2^{(k-1)} + \exp{(\omega_2)}}$$

A new input $u^{(k)} \equiv \mu_1^{(k)}$ generates a prediction error $\delta_1^{(k)}$ and the model updates and generates a new prediction as follows:

$$\delta_1^{(k)} \equiv \mu_1^{(k)} - \hat{\mu}_1^{(k)}$$

$$\pi_2^{(k)} = \hat{\pi}_2^{(k)} + \frac{1}{\hat{\pi}_1^{(k)}}$$

$$\mu_2^{(k)} = \mu_2^{(k-1)} + \frac{1}{\pi_2^{(k)}}\delta_1^{(k)}$$

$$\hat{\mu}_1^{(k+1)} \equiv s\left(\mu_2^{(k)}\right)$$

The subject's response $y^{(k)}$ (i.e. where on the continuous or Likert scale they responded) is determined by $\hat{\mu}_1^{(k+1)}$ and the precision of the response model's beta distribution ν.

Updates to x_2 are driven by the product of the prediction errors from Bayesian updating explained above and a learning rate determined by parameter ω_2: changes in x_2 from trial to trial occur according to a Gaussian random walk whose variance depends upon the static parameter ω_2: $p\left(x_2^{(k+1)}\right) \sim \mathcal{N}\left(x_2^{(k)}, \exp(\omega_2)\right)$.

The parameters ω_2 and ν were estimated individually for each participant. The (weakly informative) prior probability distributions for their values were: $\omega_2 \sim N(-3{,}16)$ and $\nu \sim N(128{,}4)$. The model's prior beliefs at the start of the sequence were fixed at $\mu_2^{(0)} = 0$ (i.e. believing each jar to be equally likely).

3) Hierarchical Gaussian Filter: 2 levels, with non-linear updating

This HGF model is exactly the same as Model 2, but with one additional parameter κ_1 which causes non-linear belief updates (see Adams et al, 2018[14]). In this model, changes in μ_2 from trial to trial occur according to two parameters: ω_2, the variance of the Gaussian random walk, and κ_1, a scaling factor that changes the size of updates when $\hat{\mu}_1 = 0.5$, or maximum

uncertainty, relative to when $\hat{\mu}_1$ is closer to 0 or 1, i.e. when the participant is more confident about either jar. Formally, the scaling occurs as:

$$\hat{\mu}_1^{(k+1)} \equiv s\left(\mu_2^{(k)}\kappa_1\right)$$

When $\kappa_1 > 1$, updating towards 1 on observing a blue bead ($u = 1$) is greatest (i.e. switching between jars becomes more likely) when $\hat{\mu}_1 < 0.3$; when $\kappa_1 < 1$, updating is comparatively far lower when $\hat{\mu}_1 < 0.3$. This means that when $\kappa_1 > 1$, the agent readily switches between jars on receiving unexpected evidence, but finds it difficult to become more confident about the current jar on receiving consistent evidence. The reverse is the case for when $\kappa_1 < 1$.

κ_1 also scales the updates for the precision and mean at second level of the model (it is fixed to 1 in Model 2), thus:

$$\pi_2^{(k)} = \hat{\pi}_2^{(k)} + \frac{\kappa_1^2}{\hat{\pi}_1^{(k)}}$$

$$\mu_2^{(k)} = \mu_2^{(k-1)} + \frac{\kappa_1}{\pi_2^{(k)}}\delta_1^{(k)}$$

The parameters κ_1, ω_2 and ν were estimated individually for each participant. The prior probability distributions for their values were: $\kappa_1 \sim N(1,1)$, $\omega_2 \sim N(-3,16)$ and $\nu \sim N(128,4)$. The model's prior beliefs at the start of the sequence were fixed at $\mu_2^{(0)} = 0$ (i.e. believing each jar to be equally likely).

4) Hierarchical Gaussian Filter: 3 levels, Autoregressive (AR1) volatility model

This HGF model used the perceptual model 'tapas_hgf_ar1_binary' and the response model 'tapas_beta_obs'. Its first two levels are exactly as described in Model 2. It also has a third level which models volatility in the contingencies, which can affect the learning rate at the level below. A relatively short sequence of 30 beads (with only one change) is too short to explore participants' estimation of volatility in detail, other than to assess whether their learning rate changes during the course of the sequence (see below).

At the top level of the model, x_3 (and its posterior estimate μ_3) encodes the phasic volatility (more properly, the log-volatility) of x_2 which determines the probability of the jar changing at any point. Parameters which affect the degree to which x_2 and x_3 can change during the experiment include m, φ, ω_3 and ω_2.

Changes in x_2 from trial to trial occur according to a Gaussian random walk whose variance depends upon both static and dynamic factors. The volatility x_3 has a dynamic influence on this learning rate (alongside the static influence of ω_2), so that in a more volatile environment one learns more quickly: $p\left(x_2^{(k+1)}\right) \sim \mathcal{N}\left(x_2^{(k)}, \exp(x_3^{(k)} + \omega_2)\right)$.

x_3 evolves according to an autoregressive (AR(1)) process controlled by three parameters: m, a level of volatility to which x_3 is attracted, φ, the rate of change of x_3 towards m, and ω_3,

the variance of the random process: $p\left(x_3^{(k+1)}\right) \sim \mathcal{N}\left(x_3^{(k)} + \varphi(m - x_3^{(k)}), \exp(\omega_3)\right)$. In effect, m describes the level of volatility in high-level beliefs that one wishes to entertain. The AR(1) process at this third level can therefore account for individual differences in updating as the sequence evolves: for example, participants who take (proportionally) longer to infer there has been a change of jar than they did to infer the correct jar at the start of the sequence, known in psychology as a 'reversal learning' impairment. The model would account for this by having a higher initial volatility estimate that subsequently declines to a new level m during the sequence.

The parameters m, φ, ω_3 and ω_2 were estimated individually for each participant. The (weakly informative) prior probability distributions for their values were standard for the HGF: $m \sim N(1,1)$, $\varphi \sim N(0.1,2)$, $\omega_3 \sim N(-6,16)$ and $\omega_2 \sim N(-2,16)$. For the response model, $\nu \sim N(128,4)$. The model's prior beliefs at the start of the sequence were fixed at $\mu_2^{(0)} = 0$ (i.e. believing each jar to be equally likely) and $\mu_3^{(0)} = 1$.

5) Finite Retrospective Inference Hidden Markov model

This model[18,19] is described in detail elsewhere: the following is a summary of its important features. It assumes that participants are using Bayesian inference to determine which of two jars is currently favoured (similar to Models 2-4), but in addition, it allows participants to update a key parameter as the sequence unfolds, rather than assuming that all parameters are fixed for the duration of the sequence (as Models 1-4 do). This parameter is *r*, the reversal probability – i.e. the probability the jars will switch on any given trial (see below).

Otherwise, the model is a standard Hidden Markov model, containing two states (corresponding to the majority red and majority blue jars). On each trial it updates the probability of these states according to adjustment rate *a* and reversal probability *r*. Adjustment rate *a* is equivalent to the cue validity, and determines the likelihood (matrix **A**) of observations given the state:

$$\mathbf{A} = \begin{bmatrix} a & 1-a \\ 1-a & a \end{bmatrix}$$

Here, the columns represent the two states (red and blue majority jars, respectively), and the rows the probabilities of observing the outcomes (red and blue beads respectively). Reversal probability *r* determines the transition (i.e. jar change) probabilities (matrix **T**) from trial to trial:

$$\mathbf{T} = \begin{bmatrix} 1-r & r \\ r & 1-r \end{bmatrix}$$

Here, the columns represent the two states (red and blue majority jars, respectively) on trial *n*, and the rows the same two states on trial *n*+1. The agent's placement of the cursor on the slider – given the probability of the states it has inferred – was parameterised by a beta distribution response model (as in Models 1-4), with precision *v*. To make the response noise measures of the same form as the decision noise in the Draws-To-Decision task – i.e.

lower values reflecting less 'noise' – we report the variance (inverse) of the response distribution, $1/\nu$.

Most models of cognition only consider inference about current states given past states: known as Bayesian 'filtering'. The optimal use of all available information, however, would also involve updating beliefs about the past given new information, known as Bayesian 'smoothing'. This is impossible for agents performing online inference, as it means they would have to store and update all their beliefs about the past. A computationally parsimonious approximation is to store beliefs about the past up to a fixed window length (of *L* trials), and only these beliefs are updated on receiving new information, known as 'fixed-lag smoothing'. This retrospective belief updating does not affect current and future beliefs (i.e. a filtering agent would make the same responses as a filtering and smoothing agent in the Probability Estimation task), however, *unless* this retrospective information is used to update parameter estimates online[19]:

For example, in the Probability Estimation beads task, the participant is uncertain both about the jar from which beads are being drawn, and about the reversal probability parameter, i.e. the probability this jar will change at any given trial. To perform better, participants may therefore update their beliefs not just about states (i.e. inference) but also parameters (i.e. learning) during the task. For example, if a participant begins the task thinking that the reversal probability *r* is around 0.2, but after 25 trials they have only seen one probable reversal, they would be wise to revise this estimate of *r* downwards, as otherwise they will overestimate the probability the jar has changed if they see an unexpected bead colour next trial. This effect would manifest as a difference in updating in the second versus the first half of the sequence (one example of such is an impairment in so-called 'reversal learning', i.e. becoming confident about the initial jar fairly quickly, but taking much longer to decide that it has changed). This also illustrates the mutual dependency between beliefs about states and beliefs about parameters: *it follows that performing fixed-lag inference about past states improves one's current parameter estimates, and thus future performance*[19].

In the Finite Retrospective Inference model, participants can update their beliefs about *r* as the sequence continues, with or without using beliefs about past states. Two parameters determine the degree to which this happens: the window length *L* over which fixed-lag smoothing is performed, and the confidence in *r*, $\Pi^{(r)}$ (a Dirichlet parameter over the transition matrix **T**). $\Pi^{(r)}$ can be seen as the number of observations about transitions that the participant has made prior to the start of the sequence, so $\Pi^{(r)}$ is inversely related to how much they are willing to update this belief henceforth. Therefore a low number (e.g. $\Pi^{(r)} = 1$) means they are very uncertain about *r*, and might revise their beliefs about it if given evidence to do so: a high number (e.g. $\Pi^{(r)} = 300$) means they are very certain about *r* and will not update it whatever happens during the sequence.

We included this model because it could potentially capture numerous possible abnormalities of inference that might relate to psychotic experiences: not just altered reversal probability or adjustment rate, but also a reluctance to update parameters in the

face of evidence, or a reduced time window (i.e. short term memory) from which evidence for parameter updating can be drawn.

The log joint probability distribution for the whole model depends on a vector of observations $o_{1:T}$, a vector of hidden states $x_{1:T}$, an initial distribution over x_0 ($\mathbf{d}$), $\mathbf{A}$, $\mathbf{T}$, and $\Pi^{(r)}$:

$$\ln p(o_{1:T}, x_{1:T}, \mathbf{T}, \mathbf{A} \big| \mathbf{d}, \Pi^{(r)}) = \sum_{i=1}^{T} \ln p(o_i|x_i, \mathbf{A}) + \sum_{i=2}^{T} \ln p(x_i|x_{i-1}, \mathbf{T}) + \ln p(\mathbf{T}|\, \Pi^{(r)}) + \ln p(\mathbf{A}) + \ln\ p(x_1|\mathbf{d})$$

Model inversion can then be performed by iterating variational update equations for the states and parameters[18,19].

Model fitting and comparison

Model estimation was performed using Matlab R2015a (Natick, Massachusetts: The MathWorks Inc). Models 1-4 were fitted using the HGF toolbox using the priors over parameters documented above. Model fitting for Model 5 was performed according to maximum likelihood, after a grid search was performed over model parameters using the following values:

Reversal probability *r*: 0.025-0.8 in gradations of 0.025

Adjustment rate *α*: 0.5-0.98 in gradations of 0.02

Window length *L*: 1, 2, 4, 8 or 16 trials

Confidence in *r* (Dirichlet parameter) $\Pi^{(r)}$: 1, 2, 3, 4, then exp(1.6-5.8) in gradations of 0.2.

Response precision ln(*v*): 0-7.4 in gradations of 0.2

Model 1 (Pearce-Hall) performed poorly and fitting failed in a majority of subjects: this is likely because it was unable to accommodate the pattern of belief updates that occur when subjects quickly switch between two underlying states but then make relatively small belief updates following this large change.

For the remaining models, we computed the BIC for each model given their *k* parameters, *n*=30 data points and maximum likelihood $\hat{L}$ according to:

$$\mathrm{BIC} = k \ln(n) - 2\ln(\hat{L})$$

We converted the BICs to an approximation of the log model evidence:

$$\mathrm{LME} \approx -\frac{1}{2}\mathrm{BIC}$$

We then performed Bayesian model comparison[20,21] using the approximate log model evidences and spm_BMS (SPM12, Wellcome Centre for Human Neuroimaging). The results are shown in Figure S2: the probability of each model performing best for any given subject

is shown on the left, and the probability of each model being the best overall, over and above chance (the protected exceedance probability) on the right. Model 5 (Finite Retrospective Inference HMM) was the winner. The second placed model (Model 3), like Model 5 but unlike the remaining models, had parameters that permitted differential updating to disconfirmatory versus confirmatory evidence. Model 5 does so because reversal probability has opposite effects on the size of these updates; Model 3 does so thanks to its belief instability parameter κ_1 – this was the winning model in a previous study of belief updating in schizophrenia[14].

Model 5 parameters

The distributions of Model 5 parameters are shown in Figure S3. The correlation between parameters is shown in Supplementary Table 1. Because most parameters were not normally distributed and could not straightforwardly be transformed into normal distributions, thresholds were used in these cases to group subjects into categories when examining these as outcomes:

Reversal probability, *r*:
As the parameter was not normally distributed and difficult to transform, the top 10% of the participants (with *r*>0.5) were grouped separately from the bottom 90% when examining this parameter as an outcome. As sensitivity analyses we also examined i) a cut-off at the 85th percentile, ii) a cut-off at the 95th percentile.

Adjustment rate, *a*:
This measure was transformed on a logarithm scale when examining this as an outcome.

Window length, *L*:
This measure was transformed on a logarithm scale to the power of 2 when examining this as an outcome.

Confidence in *r* (Dirichlet parameter), $\Pi^{(r)}$:
This distribution contains a large minority of subjects who are very uncertain about *r* (i.e. $\Pi^{(r)}$ is close to 1, around 33%), a small number of subjects who do not update their belief about r at all ($\Pi^{(r)}$ >300, around 6%), and the rest are intermediate. Given the published literature on belief updating and psychosis, one could hypothesise that psychotic experiences could be associated with either end of this spectrum: i.e., either maximal or minimal uncertainty about the reversal probability. Therefore, a three-category variable was used reflecting high, middle and low parameter uncertainty (with the middle category used as the baseline).

Response noise, *1/ v*:
The response precision parameter (*v*) was inverted for subsequent analysis to make it compatible with the decision noise parameter from the DTD task (i.e. for both parameters, higher values mean greater ‘noise’). It was then transformed on a logarithm scale when examining this parameter as an outcome.

Confounding Variables

Maternal education was collected at 32 weeks of pregnancy based on achievement of 'O' levels; subject-specific qualifications that were generally obtained at age 16 years (the minimal school leaving age from 1974 in England). Household income was based on equivalised income reported between 33-47 months of age separated into quintiles.

Polygenic risk scores (PRS) for schizophrenia were generated in previous work by Jones and colleagues[22] as the weighted mean number of disorder risk alleles in approximate linkage equilibrium. Scores were standardized using Z-score transformation. Risk alleles were defined as those associated with case-status in recent large consortia analyses of schizophrenia (40,675 cases and 64,643 controls)[23]. Risk alleles were defined as those associated at $p < 0.05$ as this threshold has previously been shown to maximally capture phenotypic variance for schizophrenia[23]. IQ was assessed at age 8 years using the Wechsler Intelligence Scale for Children (3rd edition). Executive functioning was assessed at age 8 years using the opposite worlds task from the Test of Everyday Attention for Childhood (TEA-Ch). Working memory was assessed at age 8 years using the WISC-III Digit Span task.

Supplementary Results

We simulated data using the winning model to show how higher r (reversal probability) relates to the behavioural measures of increased updating to disconfirmatory evidence and reduced updating to consistent evidence (Figure S4). It can be seen that even a small change in r – from 0.05 (red line) to 0.075 (blue line) – leads to an increase in updating to 'disconfirmatory' beads 4 and 27, for example, but after consistent evidence for one jar (e.g. from beads 7-13 or 21-26), the blue line is further from certainty about the jar. Other parameter values in these simulations were a = 0.7, L = 1, $\Pi^{(r)}$ = 320 (i.e. no updating of r occurs during the task).

We performed a parameter recovery analysis for the winning Probability Estimates task model because it has a relatively large number of parameters (5) given the number of data points (30). We simulated 100 datasets using parameter values drawn randomly from the ranges observed in the sample (Figure S3). We then estimated parameters from these simulated data, and computed the Spearman correlations between the ground truth and recovered parameter values (Figure S5). Three parameters were estimated with great accuracy (all ρ>0.9) – r (reversal probability), a (adjustment rate) and v (response precision): note that r and v were the only parameters associated with psychotic experiences and trauma. Two parameters could not be recovered accurately (both ρ<0.2) – $\Pi^{(r)}$ (confidence in r) and L (window length) – and likely require longer data sequences to be estimated reliably.

We performed an additional sensitivity analysis for the Probability Estimates task modelling results by checking whether subjects who appeared to make some irrational or 'outlier' responses might distort the associations between model parameters and other variables. Outlier subjects were defined as those whose responses showed minimal variability (i.e., all

30 responses were between 0.45 and 0.55) and those who made at least two 'impossible' responses, of being certain that the jar was the opposite colour to the last bead they had seen. 287/3611 subjects (8%) fulfilled at least one of these criteria.

Median parameter values in the 'outlier' and 'non-outlier' groups (respectively) were:
r = 0.10 versus r = 0.30 (whole group r = 0.275)
a = 0.66 versus a = 0.64 (whole group a = 0.64)
L = 2 versus L = 4 (whole group L = 4)
$\Pi^{(r)}$ = 3 versus $\Pi^{(r)}$ = 3 (whole group $\Pi^{(r)}$ = 3)
$1/v$ = 0.45 versus $1/v$ = 0.33 (whole group $1/v$ = 0.33)

If more liberal criteria for outliers were used (of all 30 responses being between 0.4 and 0.6, and only one or more 'impossible' responses), 513/3611 subjects (14%) were outliers. Median parameter values in the 'outlier' and 'non-outlier' groups (respectively) were:
r = 0.175 versus r = 0.30 (whole group r = 0.275)
a = 0.66 versus a = 0.64 (whole group a = 0.64)
L = 2 versus L = 4 (whole group L = 4)
$\Pi^{(r)}$ = 2 versus $\Pi^{(r)}$ = 3 (whole group $\Pi^{(r)}$ = 3)
$1/v$ = 0.42 versus $1/v$ = 0.31 (whole group $1/v$ = 0.33)

Overall, it is clear that removing subjects with outlier responses makes little difference to the median parameter values, even using a liberal threshold. Crucially, given reversal probability in the outlier subjects was consistently estimated to be lower than in the remaining subjects, outliers are unlikely to explain any association between psychotic experiences and a *higher* reversal probability. We also performed model comparison in the outlier and non-outlier groups separately: in both cases, Model 5 won convincingly.

We also assessed associations between the parameters of the runner-up model, Model 3, and psychotic experiences, as a previous modelling study of patients with schizophrenia[14] found that the belief instability parameter κ_1 and response precision (similar to inverse decision noise) v were higher and lower (respectively) in these individuals in two separate datasets. We did not find any significant associations between these parameters (or the two other parameters) and psychotic experiences in this population cohort, however.

Missing data

In those with observed data on the DTD task and psychotic experiences, the proportion of missing data was as follows:

Variable	% missing data
Sex	0%
IQ	22%
Working memory	24%
Attention	20%
Social class	20%
Maternal education	10%
Income	17%
Crowding index	18%
Schizophrenia PRS	32%
Trauma	7%

Figure S1: Flow chart of participant inclusion

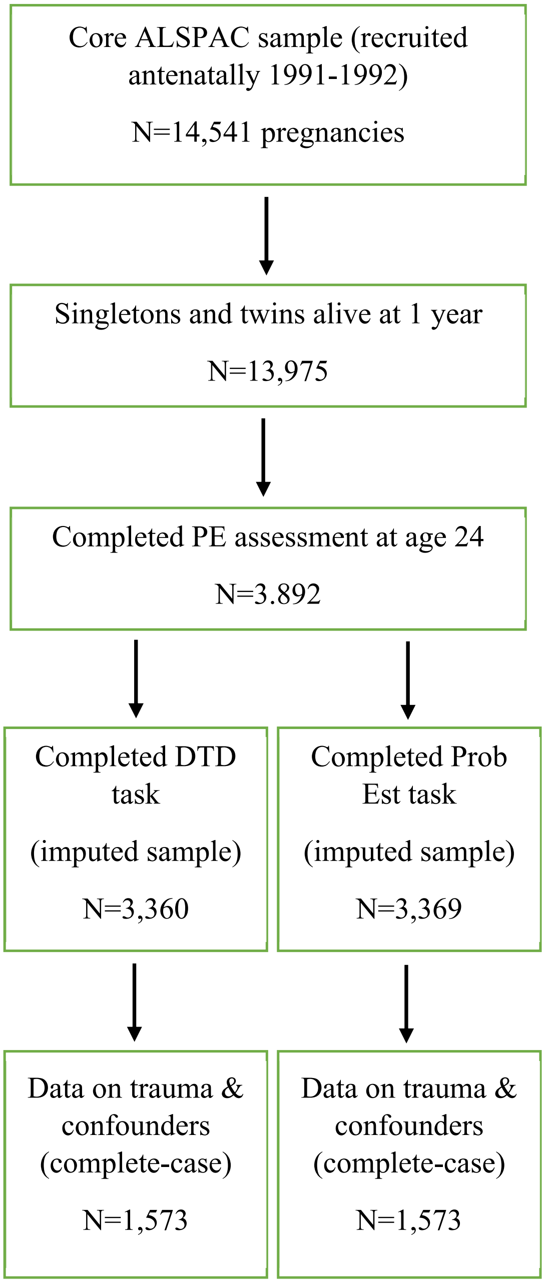

Figure S2: Model comparison for Probability Estimation task models

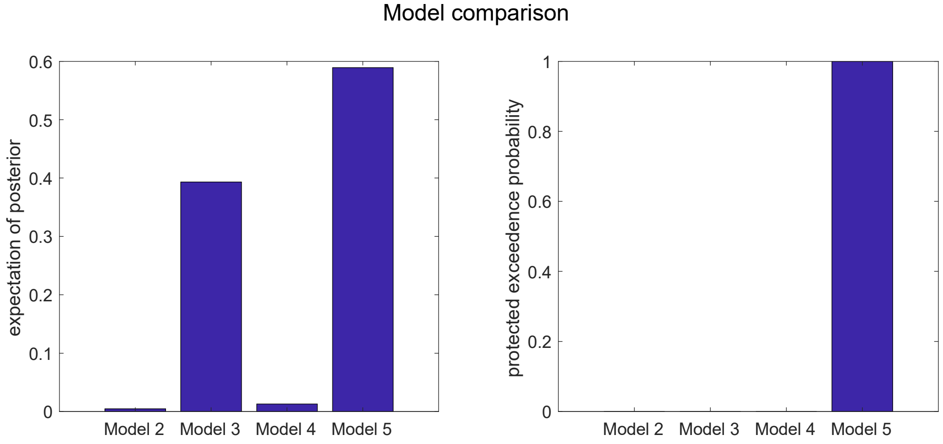

This figure shows the model comparison results for four Probability Estimation models using Bayesian Model Selection (Model 1 – Pearce-Hall – failed in many subjects and was therefore omitted from the analysis). The models are:

Model 2: Hierarchical Gaussian Filter: 2 levels

Model 3: Hierarchical Gaussian Filter: 2 levels, with non-linear updating

Model 4: Hierarchical Gaussian Filter: 3 levels, with autoregressive (AR1) volatility model

Model 5: Finite Retrospective Inference Hidden Markov model

The left plot shows the expectation of the posterior for each model, i.e. the probability that a given model will be superior for any randomly selected subject. The right plot shows the protected exceedance probability of each model, i.e. the probability that one model is more frequent than any other model, above and beyond chance. Model 5 is the winning model, with Model 3 the runner-up.

Figure S3: Distribution of belief-updating indices and derivation of categorical and binary measures

Draws to decision task

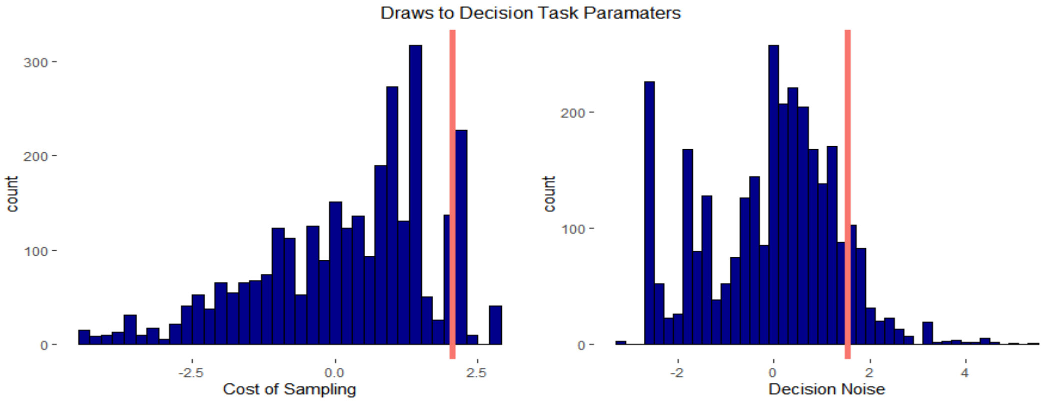

Probability Estimation Task

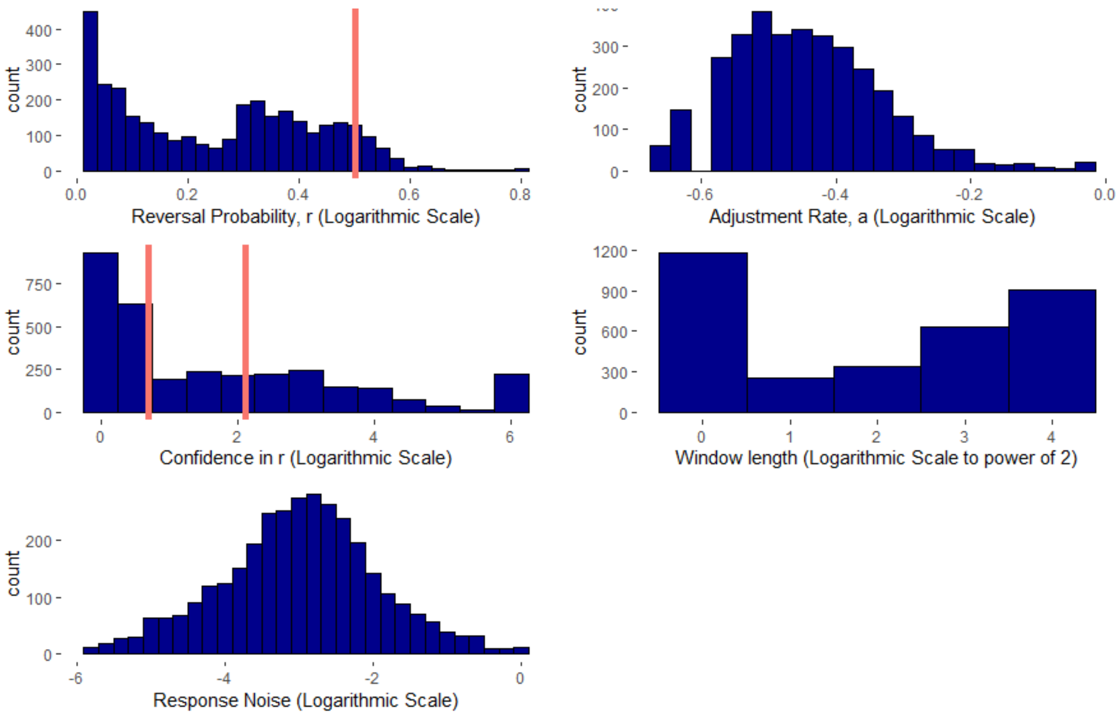

Note: Red lines denote partition of data for categorical or binary measures.

Figure S4: Simulations showing the effects of increasing *r* on inference in the Probability Estimates task

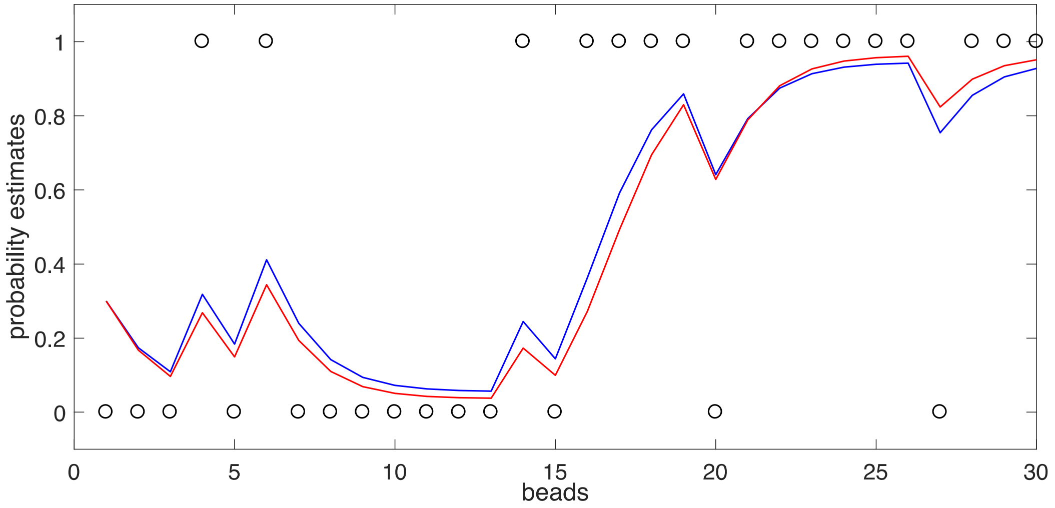

Note: The red line denotes a simulation in which $r = 0.05$, the blue line denotes a simulation in which $r = 0.075$. This is a small change but the effects on inference are clear: there is increased updating to disconfirmatory evidence, but reduced updating to consistent evidence.

Figure S5: Parameter recovery analysis – Spearman correlations between parameter values used to simulate data (x axes) and parameter values estimated from those data (y axes)

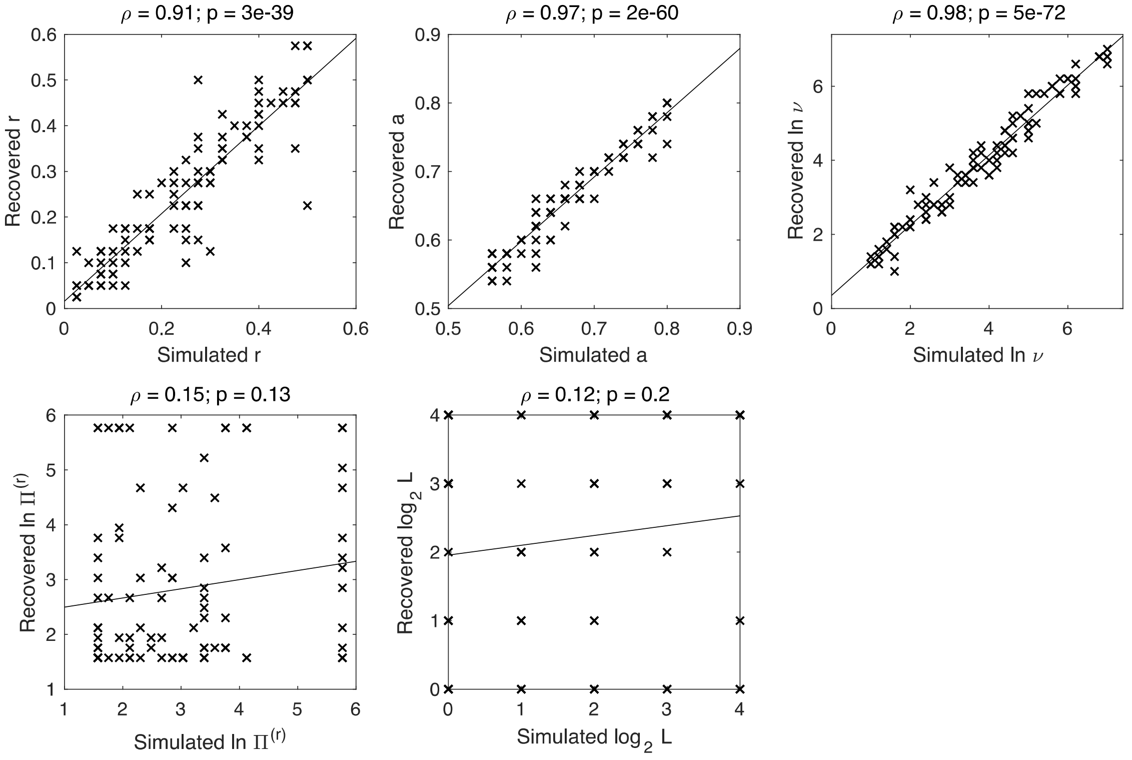

Table S1: Correlation between belief-updating behavioural measures and computational parameters

**Measures and parameters**	(1)	(2)	(3)	(4)	(5)	(6)	(7)	(8)	(9)
**Draws to Decision Task**									
Average Draws to Decision (1)	-								
High Cost of Sampling (2)	-0.49	-							
High Decision Noise (3)	-0.28	0.19	-						
**Probability Estimation Task**									
Disconfirmatory updating (4)	-0.30	0.10	0.18	-					
High reversal probability (5)	-0.13	0.10	0.26	0.55	-				
Adjustment Rate (6)	-0.28	0.18	0.05	0.61	0.25	-			
Window Length (7)	0.04	-0.02	-0.01	0.14	-0.04	-0.24	-		
Confidence in *r* (8)	-0.07	0.04	0.01	-0.06	0.01	0.05	0.11	-	
Response Noise (9)	-0.24	-0.02	0.15	0.12	0.06	0.25	-0.02	-0.01	-

Note: According to the variable type, tetrachoric correlation (binary), polychoric (categorical) and point-biserial correlation (binary and continuous) analyses were carried out.

Table S2: Mediation analysis of trauma (exposure to 3+ trauma types), higher decision noise (draws to decision task) and PEs at age 24 years

	Odds ratio	95% CI
Controlled direct effect	3.49	2.08, 4.89
Natural indirect effect	1.03	0.99, 1.07
Marginal total effects	3.58	2.13, 5.03

Note: all estimations adjusted for maternal education; imputed sample (n=3360)

Table S3: Belief-updating indices and psychotic disorder at age 24 years[1]

	Unadjusted Model			Adjusted Model[2]		
Belief-updating indices	Odds ratio	95% CI	P-value	Odds ratio	95% CI	P-Value
**Draws to Decision task**						
Average DTD	0.82	0.71, 0.95	0.008	0.86	0.74, 1.00	0.057
Cost of sampling	0.99	0.85, 1.16	0.937	1.00	0.85, 1.17	0.999
Decision noise - linear	1.24	1.05, 1.46	<0.001	1.17	0.99, 1.39	0.008
Decision noise - quadratic	1.07	1.01, 1.14		1.07	1.00, 1.14	
**Probability Estimation Task**						
Contrary updating	1.06	0.94, 1.21	0.328	1.02	0.89, 1.17	0.804
Reversal probability	2.38	0.65, 8.73	0.191	1.87	0.48, 7.27	0.367
Adjustment rate	0.80	0.04, 18.15	0.890	0.65	0.03, 15.16	0.790
Low confidence	1.31	0.67, 2.54	0.431	1.31	0.67, 2.56	0.436
High confidence	1.39	0.70, 2.79	0.349	1.40	0.69, 2.83	0.351
Window length	1.02	0.89, 1.18	0.766	1.01	0.88, 1.17	0.876
Response noise	1.19	0.95, 1.50	0.130	1.07	0.84, 1.36	0.578

[1]Imputed sample (DTD task n=3360; Probability Estimation task n=3369) [2]Adjusted for working memory, IQ, executive functioning, sex, social class, crowded living conditions, income, maternal education, genetic risk for schizophrenia, and exposure to trauma

Table S4: Bivariate analysis of belief-updating indices, hallucinations and delusions[1]

Belief-updating indices[2]	Hallucinations			Delusions			Comparison P value[4]
	Odds Ratio[3]	95% CI	P-value	Odds Ratio[2]	95% CI	P-value	
**Draws to Decision Task**							
Average draws to decision	0.95	0.89, 1.01	0.081	0.99	0.93, 1.06	0.905	0.288
Cost of Sampling	1.04	0.98, 1.11	0.179	0.96	0.90, 1.02	0.197	0.035
Decision Noise – linear	1.00	0.94, 1.06	0.112	1.03	0.96, 1.11	0.118	0.891[5]
Decision noise - quadratic	1.03	1.00, 1.06		1.03	1.00, 1.06		
**Probability Estimation Task**							
Contrary updating	1.00	0.95, 1.06	0.991	0.99	0.93, 1.06	0.837	0.853
Expectation of reversal	0.92	0.54, 1.56	0.746	1.34	0.75, 2.41	0.321	0.275
Adjustment Rate	1.08	0.32, 3.63	0.895	0.59	0.15, 2.39	0.462	0.462
Low confidence	1.08	0.84, 1.40	0.546	1.07	0.81, 1.42	0.626	0.947
High confidence	1.19	0.90, 1.55	0.217	1.02	0.76, 1.38	0.907	0.387
Inference Length	1.02	0.96, 1.07	0.548	1.01	0.95, 1.08	0.666	0.926
Response Noise	1.08	0.95, 1.14	0.390	1.03	0.93, 1.15	0.535	0.901

[1] Imputed sample (3360 for DTD task, n=3244 for response noise, n=3369 for rest of probability estimation task); [2]Average DTD and Contrary updating are behavioural measure – all others computational; [3]Adjusted for working memory, IQ, executive functioning, sex, social class, crowded living conditions, income, trauma, and genetic risk for schizophrenia; [4]Wald test p-value; [5]Wald test based on binary measure of high decision noise for modelling purposes

Table S5: Multivariable analysis of belief-updating indices and depression, anxiety and psychotic experiences

	Depression			PEs			Anxiety			Dep vs Anx	Anx vs PEs	Dep vs PEs
Belief-updating indices[1,2]	Odds Ratio	95% CI	P value	Odds Ratio	95% CI	P value	Odds Ratio	95% CI	P value	P value	P value	P value
**Draws to Decision Task**												
Draws to decision	1.01	0.96, 1.05	0.748	0.97	0.92, 1.02	0.295	1.03	1.00, 1.07	0.079	0.245	0.048	0.295
High cost of Sampling	1.00	0.96, 1.05	0.844	0.98	0.93, 1.03	0.411	0.98	0.94, 1.02	0.396	0.376	0.877	0.434
High decision Noise	0.96	0.76, 1.20	0.697	1.26	1.00, 1.60	0.051	1.05	0.86, 1.27	0.633	0.448	0.211	0.080
**Probability Estimation Task**												
Contrary updating	0.99	0.94, 1.03	0.477	1.01	0.97, 1.06	0.546	0.99	0.96, 1.03	0.685	0.742	0.447	0.336
High expectation of reversal	0.98	0.67, 1.43	0.911	1.25	0.75, 1.95	0.336	1.00	0.72, 1.40	0.991	0.910	0.423	0.400
Adjustment Rate	1.04	0.44, 2.46	0.928	0.91	0.33, 2.59	0.869	0.98	0.47, 2.20	0.968	0.960	0.870	0.848
Low confidence	1.15	0.96, 1.38	0.131	1.04	0.84, 1.30	0.701	1.07	0.91, 1.26	0.393	0.474	0.834	0.485
High Confidence	1.14	0.94, 1.39	0.181	1.06	0.85, 1.23	0.580	1.12	0.94, 1.31	0.217	0.809	0.762	0.644
Inference Length	1.00	0.96, 1.04	0.879	1.01	0.96, 1.06	0.632	0.99	0.96, 1.03	0.709	0.651	0.526	0.779
Decision Noise	0.99	0.93, 1.06	0.845	1.05	0.97, 1.14	0.193	0.99	0.93, 1.05	0.813	0.986	0.218	0.241

[1] Imputed sample (3425 for DTD task, n=3307 for response noise, n=3434 for rest of probability estimation task); [2]Adjusted for working memory, IQ, executive functioning, sex, social class, crowded living conditions, income, and genetic risk for schizophrenia

Table S6: Sensitivity analyses of belief updating and psychotic outcomes using percentile cut-offs for non-normal continuous measures[1]

	Psychotic experiences			Psychotic disorder		
	Odds Ratio[2]	95% CI	P-Value	Odds Ratio[2]	95% CI	P-Value
**Draws to Decision Task**						
Cost of sampling (85th percentile)	0.86	0.52, 1.42	0.552	0.89	0.45, 1.77	0.743
Cost of sampling (90th percentile)	1.05	0.60, 1.84	0.860	0.78	0.33, 1.85	0.578
Cost of sampling (95th percentile)	0.68	0.27, 1.71	0.410	1.13	0.40, 3.20	0.822
Decision Noise (85th percentile)	1.38	0.89, 2.12	0.148	1.50	0.84, 2.69	0.168
Decision Noise (90th percentile)	1.62	1.00, 2.62	0.048	1.87	0.99, 3.53	0.054
Decision Noise (95th percentile)	1.79	0.97, 3.29	0.063	2.22	1.01, 4.86	0.047
**Probability Estimation Task**						
Reversal probability (85th percentile)	1.74	1.12, 2.69	0.013	2.30	1.32, 4.04	0.004
Reversal probability (90th percentile)	1.45	0.84, 2.51	0.180	1.91	0.96, 3.77	0.064
Reversal probability (95th percentile)	1.72	0.90, 3.29	0.101	2.38	1.07, 5.27	0.033

[1]Imputed sample (DTD task n=3360; Probability Estimation task n=3369); [2]Adjusted for working memory, IQ, executive functioning, sex, social class, crowded living conditions, income, maternal education, and genetic risk for schizophrenia

Table S7: Sensitivity analyses of trauma and belief updating using different percentile cut-offs for non-normal continuous variables[1]

Outcome	Odds Ratio[2]	95% CI	P-Value
**Draws to Decision Task**			
Cost of sampling (85th percentile)	1.03	0.94, 1.12	0.555
Cost of sampling (95th percentile)	1.05	0.90, 1.22	0.526
Decision Noise (85th percentile)	1.21	1.10, 1.33	<0.001
Decision Noise (95th percentile)	1.28	1.09, 1.50	0.002
**Probability Estimation Task**			
Reversal probability (85th percentile)	0.97	0.88, 1.07	0.489
Reversal probability (95th percentile)	1.15	0.98, 1.35	0.079

[1]Imputed sample (DTD task n=3360; Probability estimation task n=3369); [2]Adjusted for sex, social class, crowded living conditions, income, maternal education, and genetic risk for schizophrenia

Non-parametric analyses (unadjusted) of continuous measures of cost of sampling, decision noise and reversal probability using Spearman correlations provided similar evidence of association between trauma and decision noise (mean p-value across 50 imputations <0.001), and not cost of sampling (mean p-value across 50 imputations = 0.416) or reversal probability (mean p-value across 50 imputations = 0.707)

Table S8: Belief-updating parameters and ordinal measure of number of frequent or distressing PEs at age 24 years[1]

	Unadjusted			Adjusted[3]		
Belief-updating indices[2]	OR	95% CI	P	OR	95% CI	P
**Draws to Decision Task**						
Average DTD	0.89	0.80, 0.99	0.029	0.93	0.84, 1.04	0.213
Cost of sampling	0.95	0.86, 1.06	0.388	0.96	0.86, 1.07	0.452
Decision noise - linear	1.11	0.99, 1.24	<0.001	1.05	0.94, 1.18	0.009
Decision noise - quadratic	1.09	1.04, 1.14		1.08	1.03, 1.14	
**Probability Estimation Task**						
Contrary updating	1.08	0.99, 1.18	0.071	1.04	0.94, 1.15	0.420
Reversal probability	1.99	0.78, 5.07	0.141	1.62	0.61, 4.32	0.313
Adjustment rate	1.43	0.16, 12.88	0.754	1.05	0.11, 9.72	0.973
Low confidence	1.13	0.71, 1.78	0.606	1.11	0.70, 1.77	0.626
High confidence	1.16	0.72, 1.89	0.548	1.13	0.69, 1.85	0.607
Inference Length	1..03	0.93, 1.14	0.572	1.02	0.92, 1.14	0.692
Response Noise	1.23	1.04, 1.45	0.020	1.12	0.94, 1.33	0.211

[1] Imputed sample (n=3114 for DTD task, n=3005 for response noise, n=3122 for rest of probability estimation task); [2]Average DTD and Contrary updating are behavioural measure – all others computational; [3]Adjusted for working memory, IQ, executive functioning, sex, social class, crowded living conditions, income, trauma and genetic risk for schizophrenia

Association between working memory / executive function and DTD/probability estimation task measures

	Working memory		Executive function	
	Correlation	p	Correlation	p
DTD	0.06	0.004	0.07	<0.001
Cost of sampling	0.03	0.08	0.03	0.09
Decision noise	-0.10	<0.001	-0.09	<0.001
Contrary updating	-0.13	<0.001	-0.07	<0.001
Reversal probability	-0.07	<0.001	-0.07	<0.001
Adjustment rate	-0.02	0.349	-0.01	0.459
Confidence	-0.01	0.451	0.02	0.210
Inference length	-0.03	0.188	0.02	0.354
Response noise	-0.12	<0.001	-0.05	0.005

Table S9: Belief-updating indices and frequent or distressing PEs at age 24 years in the past 12 months in complete-case sample[1]

	Unadjusted Model			Adjusted Model[2]		
Belief-updating indices	Odds ratio	95% CI	P-value	Odds ratio	95% CI	P-Value
**Draws to Decision task**						
Average DTD	0.89	0.75, 1.06	0.186	0.94	0.79, 1.12	0.465
Cost of sampling	0.92	0.77, 1.09	0.316	0.91	0.77, 1.08	0.296
Decision noise - linear	1.08	0.91, 1.27	0.012	1.03	0.87, 1.22	0.107
Decision noise - quadratic	1.09	1.02, 1.18		1.10	1.01, 1.18	
**Probability Estimation Task**						
Contrary updating	0.98	0.83, 1.17	0.850	0.93	0.78, 1.12	0.465
Reversal probability	1.30	0.28, 6.00	0.740	1.03	0.21, 4.93	0.973
Adjustment rate	0.09	0.00, 4.20	0.217	0.06	0.00, 3.16	0.166
Low confidence	1.82	0.79, 4.19	0.159	1.89	0.82, 4.38	0.136
High confidence	1.77	0.74, 4.22	0.201	1.82	0.76, 4.38	0.181
Window length	1.06	0.90, 1.24	0.460	1.06	0.90, 1.24	0.477
Response noise	1.07	0.83, 1.38	0.622	1.02	0.78, 1.33	0.901

[1]N=1573; [2]Adjusted for working memory, IQ, executive functioning, sex, social class, crowded living conditions, income, maternal education, genetic risk for schizophrenia, and exposure to trauma

Table S10: Association between exposure to trauma and belief-updating parameters (complete-case sample)

Belief-updating parameters	Effect	Unadjusted			Adjusted[1]		
		Effect Size	95% CI	P-Value	Effect Size	95% CI	P-Value
**Draws to Decision Task**							
Draws to decision	β	-0.08	-0.014, -0.01	0.027	-0.05	-0.11, 0.02	0.149
High cost of sampling	OR	0.98	0.85, 1.13	0.789	0.97	0.85, 1.11	0.674
High decision noise	OR	1.21	1.05, 1.39	0.009	1.17	1.03, 1.34	0.020
**Probability Estimation Task**							
Contrary updating	β	0.01	-0.01, 0.04	0.334	0.01	-0.02, 0.03	0.517
High reversal probability	OR	1.00	0.83, 1.20	0.992	0.96	0.81, 1.13	0.604
Adjustment rate	β	0.00	-0.00, 0.01	0.118	0.00	-0.00, 0.01	0.110
Low confidence	RRR	1.02	0.91, 1.15	0.738	1.04	0.94, 1.16	0.445
High confidence	RRR	1.00	0.89, 1.13	0.976	1.02	0.91, 1.14	0.691
Inference length	OR	0.96	0.89, 1.04	0.293	0.95	0.89, 1.03	0.208
Response noise	β	0.04	-0.01, 0.08	0.119	0.03	-0.02, 0.07	0.237

[1]Adjusted for sex, income, crowding, social class, maternal education and genetic risk for schizophrenia

Supplementary References

1. Croft J, Heron J, Teufel C, et al. Association of Trauma Type, Age of Exposure, and Frequency in Childhood and Adolescence With Psychotic Experiences in Early Adulthood. *JAMA Psychiatry.* 2019;76(1):79-86.
2. Addington J, Cadenhead KS, Cannon TD, et al. North American Prodrome Longitudinal Study: a collaborative multisite approach to prodromal schizophrenia research. *Schizophr Bull.* 2007;33(3):665-672.
3. Yung AR, Yuen HP, McGorry PD, et al. Mapping the onset of psychosis: the Comprehensive Assessment of At-Risk Mental States. *Aust N Z J Psychiatry.* 2005;39(11-12):964-971.
4. Sullivan SA, Kounali D, Cannon M, et al. A Population-Based Cohort Study Examining the Incidence and Impact of Psychotic Experiences From Childhood to Adulthood, and Prediction of Psychotic Disorder. *The American Journal of Psychiatry.* 2020:appiajp201919060654.
5. Westfall J, Nichols TE, Yarkoni T. Fixing the stimulus-as-fixed-effect fallacy in task fMRI. *Wellcome Open Res.* 2016;1:23.
6. Dudley R, Taylor P, Wickham S, Hutton P. Psychosis, delusions and the “jumping to conclusions” reasoning bias: a systematic review and meta-analysis. *Schizophrenia bulletin.* 2015:sbv150.
7. Phillips LD, Edwards W. Conservatism in a simple probability inference task. *J Exp Psychol.* 1966;72(3):346-354.
8. Ross, McKay R, Coltheart M, Langdon R. Jumping to Conclusions About the Beads Task? A Meta-analysis of Delusional Ideation and Data-Gathering. *Schizophrenia Bulletin.* 2015;41(5):1183-1191.
9. Ermakova AO, Gileadi N, Knolle F, et al. Cost Evaluation During Decision-Making in Patients at Early Stages of Psychosis. *Computational Psychiatry.* 2018;3:18-39.
10. Moutoussis M, Bentall RP, El-Deredy W, Dayan P. Bayesian modelling of Jumping-to-Conclusions bias in delusional patients. *Cogn Neuropsychiatry.* 2011;16(5):422-447.
11. Averbeck BB, Evans S, Chouhan V, Bristow E, Shergill SS. Probabilistic learning and inference in schizophrenia. *Schizophr Res.* 2011;127(1-3):115-122.
12. Banca P, Lange I, Worbe Y, et al. Reflection impulsivity in binge drinking: behavioural and volumetric correlates. *Addict Biol.* 2016;21(2):504-515.
13. Moutoussis M, Hopkins AK, Dolan RJ. Hypotheses About the Relationship of Cognition With Psychopathology Should be Tested by Embedding Them Into Empirical Priors. *Front Psychol.* 2018;9:2504.
14. Adams RA, Napier G, Roiser JP, Mathys C, Gilleen J. Attractor-like dynamics in belief updating in schizophrenia. *Journal of Neuroscience.* 2018:3163-3117.
15. Peters E, Garety P. Cognitive functioning in delusions: A longitudinal analysis. *Behaviour Research and Therapy.* 2006;44(4):481-514.
16. Pearce JM, Hall G. A model for Pavlovian learning: variations in the effectiveness of conditioned but not of unconditioned stimuli. *Psychol Rev.* 1980;87(6):532-552.
17. Mathys C, Daunizeau J, Friston KJ, Stephan KE. A bayesian foundation for individual learning under uncertainty. *Front Hum Neurosci.* 2011;5:39.

18. FitzGerald THB, Hammerer D, Friston KJ, Li SC, Dolan RJ. Sequential inference as a mode of cognition and its correlates in fronto-parietal and hippocampal brain regions. *PLoS Comput Biol.* 2017;13(5):e1005418.
19. FitzGerald THB, Penny WD, Bonnici HM, Adams RA. Retrospective Inference as a Form of Bounded Rationality, and Its Beneficial Influence on Learning. *Frontiers in Artificial Intelligence.* 2020;3.
20. Rigoux L, Stephan KE, Friston KJ, Daunizeau J. Bayesian model selection for group studies - revisited. *Neuroimage.* 2014;84:971-985.
21. Stephan KE, Penny WD, Daunizeau J, Moran RJ, Friston KJ. Bayesian model selection for group studies. *Neuroimage.* 2009;46(4):1004-1017.
22. Jones HJ, Stergiakouli E, Tansey KE, et al. Phenotypic Manifestation of Genetic Risk for Schizophrenia During Adolescence in the General Population. *JAMA Psychiatry.* 2016;73(3):221-228.
23. Pardinas AF, Holmans P, Pocklington AJ, et al. Common schizophrenia alleles are enriched in mutation-intolerant genes and in regions under strong background selection. *Nat Genet.* 2018;50(3):381-389.
